# Large-Scale Polymorphism Analysis of Dog Leukocyte Antigen Class I and Class II Genes (*DLA-88*, *DLA-12/88L* and *DLA-DRB1*) and Comparison of the Haplotype Diversity between Breeds in Japan

**DOI:** 10.3390/cells12050809

**Published:** 2023-03-06

**Authors:** Jiro Miyamae, Masaharu Okano, Fumihiko Katakura, Jerzy K. Kulski, Tadaaki Moritomo, Takashi Shiina

**Affiliations:** 1 Faculty of Veterinary Medicine, Okayama University of Science, 1-3 Ikoino-oka, Imabari 794-8555, Japan; 2 Department of Legal Medicine, Nihon University School of Dentistry, 1-8-13 Kanda-Surugadai, Chiyoda-ku, Tokyo 101-8310, Japan; 3 Department of Veterinary Medicine, College of Bioresource Science, Nihon University, 1866 Kameino, Fujisawa 252-0880, Japan; 4 Department of Molecular Life Science, Division of Basic Medical Science and Molecular Medicine, Tokai University School of Medicine, 143 Shimokasuya, Isehara 259-1143, Japan

**Keywords:** dog, dog leukocyte antigen (DLA), polymorphism, haplotype diversity

## Abstract

Polymorphisms of canine leukocyte antigen (DLA) class I (*DLA-88* and *DLA-12/88L*) and class II (*DLA-DRB1*) genes are important for disease susceptibility studies, but information on the genetic diversity among dog breeds is still lacking. To better elucidate the polymorphism and genetic diversity between breeds, we genotyped *DLA-88*, *DLA-12/88L*, and *DLA-DRB1* loci using 829 dogs of 59 breeds in Japan. Genotyping by Sanger sequencing identified 89, 43, and 61 alleles in *DLA-88*, *DLA-12*/*88L*, and *DLA-DRB1* loci, respectively, and a total of 131 *DLA-88*–*DLA-12/88L*–*DLA-DRB1* haplotypes (88-12/88L-DRB1) were detected more than once. Of the 829 dogs, 198 were homozygotes for one of the 52 different 88-12/88L-DRB1 haplotypes (homozygosity rate: 23.8%). Statistical modeling suggests that 90% of the DLA homozygotes or heterozygotes with one or other of the 52 different 88-12/88L-DRB1 haplotypes within somatic stem cell lines would benefit graft outcome after 88-12/88L-DRB1-matched transplantation. As previously reported for DLA class II haplotypes, the diversity of 88-12/88L-DRB1 haplotypes varied remarkably between breeds but was relatively conserved within most breeds. Therefore, the genetic characteristics of high DLA homozygosity rate and poor DLA diversity within a breed are useful for transplantation therapy, but they may affect biological fitness as homozygosity progresses.

## 1. Introduction

The major histocompatibility complex (MHC) molecules play important roles in inducing acquired immunity by presenting peptides derived from foreign antigens, such as germs and viruses that T cells recognize as non-self, resulting in the elimination of these antigens. The MHC molecules are classified into class I (MHC-I) and class II (MHC-II), and regulate self- and non-self discrimination in immunity by presenting antigen peptides to CD8^+^ and CD4^+^ T cells, respectively [1,2]. The MHC genes encoding MHC-I and MHC-II molecules are composed of multigene families; each of them is extremely polymorphic in many animals. For example, so far, more than 34,000 human leukocyte antigen (HLA) alleles have been identified and reported in the IPD-IMGT database (https://www.ebi.ac.uk/ipd/imgt/hla/ (accessed on 28 November 2022)). In addition, specific HLA alleles associated with susceptibility or resistance to various diseases [3,4,5] and matching of HLA polymorphisms between donor and recipient in transplantation are important factors for suppressing alloimmune responses [6,7,8,9].

The domesticated dog (*Canis lupus familiaris*) is one of the major companion animals of humans that also is used for biomedical research, such as on the pathobiology of cancers and autoimmune diseases, whose clinical phenotypes (presentations) are similar to those in humans, and in transplantation studies as a preclinical model [10,11]. A draft of the dog genome sequence was determined in the early 2000s, and the dog leukocyte antigen (DLA) loci were located on two chromosome (chr) segments, chr 12 and chr 18 [12,13]. Overall, three DLA class I (DLA-I) loci (*DLA-88*, *DLA-12*, and *DLA-64*) and four DLA class II (DLA-II) loci (*DLA-DRA*, *DLA-DRB1*, *DLA-DQA1*, and *DLA-DQB1*) were mapped on chr 12, and one divergent DLA-I locus *DLA-79* was mapped on chr 18.

To date, 173 DLA-I and 297 DLA-II alleles have been identified and released by the Canine MHC Nomenclature Committee into the IPD-MHC database (https://www.ebi.ac.uk/ipd/mhc/group/DLA/ (accessed on 28 November 2022)). Of the 173 DLA-I alleles identified in several polymorphism studies using more than 500 dogs in total, 139, 17, 9, and 8 alleles were identified in the *DLA-88*, *DLA-12*, *DLA-64*, and *DLA-79* loci, respectively [14,15,16,17,18]. In contrast, the polymorphism analyses of the class II region, using more than 10,000 dogs of over 200 breeds, identified 1, 181, 30, and 86 alleles in *DLA-DRA*, *DLA-DRB1*, *DLA-DQA1*, and *DLA-DQB1* loci, respectively [19,20,21,22]. Genetic diversity of the DLA-II haplotypes with linked *DLA-DRB1*, *DLA-DQA1*, and *DLA-DQB1* alleles has been analyzed extensively in various dog breeds. Kennedy et al. reported that DLA polymorphisms are relatively limited within a dog breed, but there are significant differences in the types and frequencies of the DLA-II haplotypes between dog breeds [20,21,22]. In addition, there are reports on *DLA-88–DLA-DRB1* haplotypes in the Beagle, *DLA-88–DLA-DRB1–DLA-DQA1–DLA-DQB1* haplotypes in the German shepherd dog and some other dog breeds [22,23,24].

Several PCR-based genotyping methods for the DLA-II genes were established, and specific DLA-II polymorphisms were associated to various diseases such as rheumatoid arthritis [25], diabetes mellitus [26,27], and chronic enteritis [28] by case-control studies in various dog breeds. In contrast, though the association between *DLA-79* polymorphisms and multiple immune-mediated diseases was reported, there were no clear associations found between polymorphisms of the other DLA-I genes and disease except for an association with pancreatic acinar atrophy in German Shepherds [23,29].

**Figure 1:**
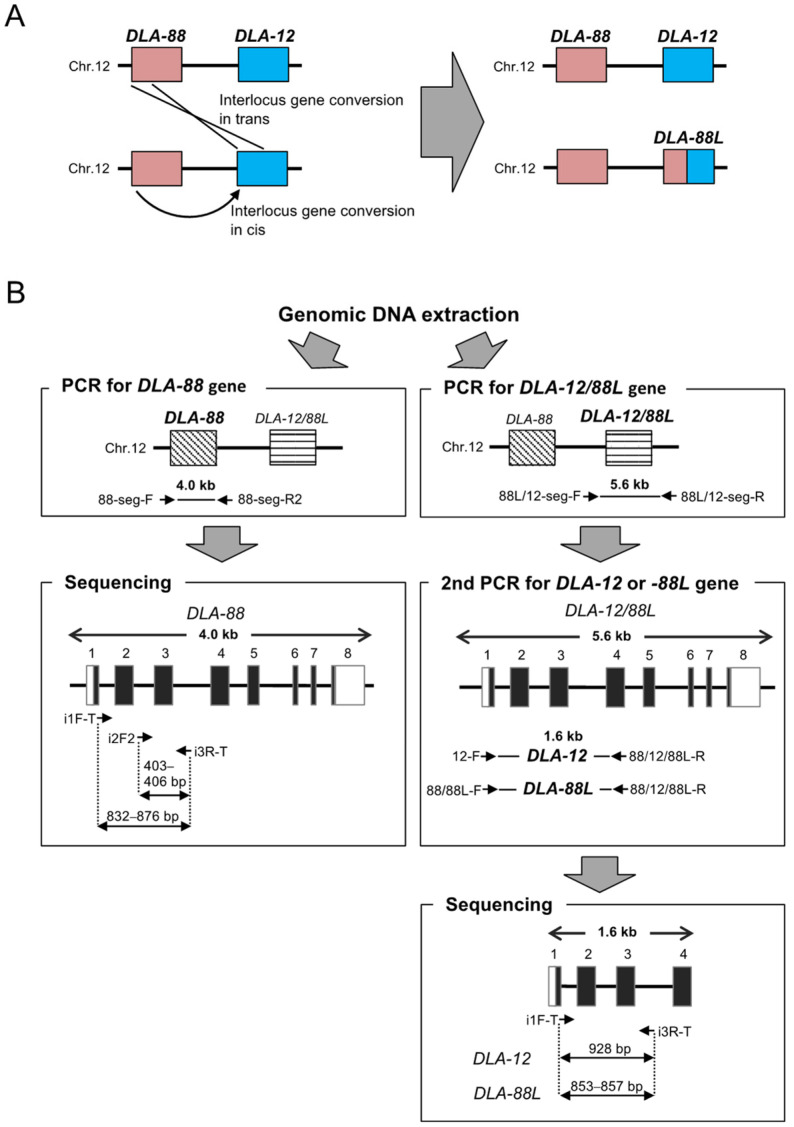
Schematic representations of haplotype structures and typing method for DLA-I genes. (**A**) Two different haplotype structures associated with *DLA-12* and *DLA-88L* locus. Interlocus gene conversion events in cis or trans between *DLA-88* and *DLA-12* are likely to be responsible for generating the *DLA-88L* locus. (**B**) Flow chart for locus-specific DLA typing method for *DLA-88*, *DLA-12*, and *DLA-88L* genes with genomic DNA. White and black-filled boxes in the chart represent the schematic location of UTR and CDS regions of a DLA gene. Arrowheads indicate the location and direction of the primers with the primer names whose detailed information is described in Supplementary Table S1.

A reason for this limited association of DLA-I polymorphisms with different dog diseases might be that a locus-specific DLA-I genotyping method for association studies had not been established properly because copy number variations of the DLA-I genes per haploid were unknown. In this regard, we recently found that a gene conversion event between *DLA-88* and *DLA-12* had generated a hybrid DLA-I locus *DLA-88L*, resulting in two distinct DLA-I haplotype structures, *DLA-88–DLA-12–DLA-64* and *DLA-88–DLA-88L–DLA-64* [18,30] (Figure 1A). We describe these two alternative haplotypes as the 88-12/88L-64 haplotypes. We also developed a polymorphism analysis method to separate the *DLA-88* and *DLA-12/88L* alleles based on their genomic structures [30]. However, no simple technology was developed to efficiently separate the *DLA-12* and *DLA-88L* alleles from each other (*DLA-12/88L*), until the present study (Figure 1B). Thus, there are few published studies on the genetic diversity of different combinations of DLA-I and DLA-II polymorphisms in various breeds. 

In this study, to characterize the intra- and inter-breed DLA diversity, including both DLA-I and DLA-II genes in various breeds, we developed a new genotyping method to separate *DLA-12* from *DLA-88L* accurately and performed polymorphism analysis of the relatively polymorphic DLA-I genes, *DLA-88* and *DLA-12/88L*, and the most polymorphic DLA-II gene, *DLA-DRB1*, using 829 dogs of 59 breeds that were collected in Japan. We also estimated three-locus *DLA-88–DLA-12/88L–DLA-DRB1* haplotypes (88-12/88L-DRB1) from the detected allele information and evaluated the genetic diversity within and between breeds based on the three-locus haplotype frequency. Furthermore, since the *DLA-88*, *DLA-12/88L*, and *DLA-DRB1* genes have the characteristics of classical MHC genes such as *HLA-A*, *HLA-B*, and *HLA-DRB1*, their DLA polymorphisms are thought to play important roles for the allo-recognition mechanism during transplantation. Hence, to evaluate the possibility of 88-12/88L-DRB1-matched transplantation using somatic stem cells in the field of veterinary medicine, we simulated statistically the proportion of recipient dogs that possibly could undergo 88-12/88L-DRB1-matched transplantation of somatic stem cells if these cells were established from the 88-12/88L-DRB1 homozygotes.

## 2. Materials and Methods

### 2.1. RNA and DNA Samples

Peripheral blood samples from 829 dogs of 59 breeds were collected from the Animal Medical Center (ANMEC) at Nihon University, Marble Veterinary Medical center, and the Nippon Veterinary and Life Science University in accordance with the guidelines for animal experiments specific to each location when the dog owner approved to use the blood for research. Of these, 403 were genotyped initially using RNA samples extracted in the previous study [18,30], and the remaining 426 were genotyped using newly extracted genomic DNA samples (Table 1). We initially genotyped RNA samples (converted to cDNA for amplification) because transcribed MHC genes and alleles are detected more easily without contamination of amplicons originating from pseudogenes or duplicated genes if primer locations crossover to at least two homologous locations. Limitations of the RNA-based genotyping method [15,18] were corrected by also genotyping genomic DNA samples.

The genomic DNA was extracted from peripheral blood mononuclear cells by using TRIzol LS Reagent (Invitrogen/Life Technologies/Thermo Fisher Scientific, Carlsbad, CA, USA) or Kaneka Easy DNA Extraction Kit version 2 (Kaneka Corporation, Hyogo, Japan) according to the manufacturer’s protocols.

### 2.2. PCR Amplification of DLA-88 and DLA-12/88L Genes

Polymorphism analysis for *DLA-88* and *DLA-12/88L* was performed using the genomic DNA from 426 dogs obtained for this study and 403 dogs that already were genotyped in our previous study [18].

The first PCR was performed independently using a specific primer set (88-seg-F and 88-seg-R2) to amplify the 4.0 kb genomic region, including *DLA-88* and using a specific primer set (88L/12-seg-F and 88L/12-seg-R) to amplify the 5.6 kb genomic region including *DLA-12/88L* (Figure 1B and Supplementary Table S1A). The composition of the PCR solution was 20 ng of DNA, 0.4 unit of KOD FX DNA polymerase (TOYOBO, Osaka, Japan), 10 uL of 2× PCR buffer, 2 mM of dNTP and 0.4 uM of each primer in 20 uL. The cycling parameter was as follows: an initial denaturation with 94 °C/1 min followed by 33 cycles of 98 °C/10 s, 63 °C/30 s, and 68 °C/4 min for *DLA-88* and 98 °C/10 s, 58 °C/30 s, and 68 °C/5 min for *DLA-12/88L*. After the PCR products were purified using ExoSAP-IT (GE Healthcare, Piscataway, NJ, USA) and diluted, the 2nd PCR to distinguish between *DLA-12* and *DLA-88L* alleles was performed using a *DLA-12* specific primer set (12-F and 88/12/88L-R) and a *DLA-88* and *DLA-88L* specific primer set (88/88L-F and 88/12/88L-R) (Supplementary Table S1B) [15,18]. The composition of the PCR solution was 1 uL of the first PCR product diluted 1000-fold, 0.4 unit of KOD FX DNA polymerase, 10 μL of 2× PCR buffer, 2 mM of dNTP, and 0.4 μL of each primer in 20 uL. The cycling parameter was as follows: an initial denaturation with 94 °C/1 min followed by 33 cycles of 98 °C/10 s, 63 °C/30 s, and 68 °C/90 s.

### 2.3. PCR Amplification of DLA-DRB1 Gene

Polymorphism analysis for *DLA-DRB1* was performed using RNA samples from the 403 dogs as templates by using *DLA-DRB1* specific primer sets (DRB1-F and DRB1-R) (Supplementary Table S1D) [31]. The cDNA samples were synthesized with the oligo-dT primer using the RevaTra Ace reverse transcriptase reaction (TOYOBO, Osaka, Japan) after DNase I treatment using 1 μg of RNA (Invitrogen/Life Technologies/Thermo Fisher Scientific, Carlsbad, CA, USA) according to the manufacturer’s protocol. The polymorphism analysis was also performed using DNA samples from the 426 dogs as templates by using *DLA-DRB1* specific primer sets (DRB1-g-F and DRB1-g-R) (Supplementary Table S1E) [32]. The composition of the PCR solution consisted of 20 ng of cDNA or genomic DNA, 0.4 units of KOD FX DNA polymerase, 10 uL of 2× PCR buffer, 2 mM of dNTP, and 0.4 uM of each primer in 20 uL. The cycling parameter was as follows: an initial denaturation with 94 °C/1 min followed by 33 cycles of 98 °C/10 s, 60 °C/30 s, and 68 °C/45 s.

### 2.4. Sanger-Sequencing

After purification of PCR products using ExoSAP-IT (GE Healthcare, Piscataway, NJ, USA), the purified PCR products were sequenced directly with Big Dye Terminator Kit Ver. 1.1 or Ver. 3.1 (Life Technologies/Thermo Fisher Scientific, Carlsbad, CA, USA) and ABI3130 genetic analyzer (Life Technologies/Thermo Fisher Scientific, Carlsbad, CA, USA). The nucleotide sequences of the PCR products for *DLA-88*, *DLA-12*, and *DLA-88L* were determined using sequencing primers i1F-T and i3R-T (Supplementary Table S1C). When the *DLA-88* allele sequences were difficult to determine due to sequence offsets by nucleotide insertions and deletions in intron 2 and/or exon 3 of the *DLA-88* alleles [30,33], additional sequencing was performed using another primer i2F2 (Figure 1B).

### 2.5. Allele Assignment and Confirmation of Novel DLA Alleles

DLA allelic sequences were assigned using Sequencher Ver. 5.0.1 DNA sequence assembly software (Gene Code Co., Ann Arbor, MI, USA) by comparing them with known *DLA-88*, *DLA-88L*, *DLA-12*, and *DLA-DRB1* allele sequences released in the GenBank (https://www.ncbi.nlm.nih.gov/genbank/ (accessed on 26 April 2022)) and the IPD-MHC Canines database (https://www.ebi.ac.uk/ipd/mhc/group/DLA/ (accessed on 26 April 2022)). Allele sequences from the Sanger sequencing data also were assigned using the MHC allele assignment software Assign ATF ver. 1.0.2.45 (Conexio, Western Australia, Australia). New alleles were confirmed by PCR and direct sequencing again. The PCR products were cloned into the pTA2 cloning vector with the TA cloning kit (TOYOBO, Osaka, Japan), and the nucleotide sequence in 4 to 8 clones per DNA sample was analyzed to avoid PCR and sequencing artifacts.

### 2.6. Nomenclature of Novel DLA Alleles

We defined the alleles amplified by using the *DLA-88* and *DLA-88L* specific primer set as the *DLA-88L* allele and the alleles amplified by using the *DLA-12* specific primer set as *DLA-12* allele in the 2nd PCR of *DLA-12/88L*. Since all the *DLA-88L* alleles reported so far in the IPD-MHC Canines database have been named “DLA-88*”, we also followed the rule for the identified *DLA-88L* novel alleles as well as all published *DLA-88* alleles. The official name of the novel allele was assigned according to the DLA nomenclature in the IPD-MHC database. Novel DLA alleles that have not been given an official allele name were named “DLA-88*nov”, “DLA-12*nov”, or “DLA-DRB1*nov” as tentative allele names.

### 2.7. Estimation of DLA-88–DLA-12/88L–DLA-DRB1 (88-12/88L-DRB1) Haplotypes

The 88-12/88L-DRB1 haplotypes for each dog, which are 88-12-DRB1 or 88-88L-DRB1, were identified and estimated manually based on genotyping data of 829 dogs as previously described [18,20]. We initially identified the 88-12/88L-DRB1 haplotypes for homozygous dogs and estimated the haplotypes for heterozygous dogs on the basis of those within the homozygous dogs. To confirm our manual haplotype estimation, we also estimated the 88-12/88L-DRB1 haplotypes in each breed by using the maximum likelihood method of the PHASE program [34].

### 2.8. Data Analysis

Calculation of expected heterozygosity (He), Hardy–Weinberg equilibrium (HWE) test, and principal component analysis (PCA) based on the 88-12/88L-DRB1 haplotype frequencies in each breed were performed by GenAlEx Ver. 6.5 [35]. In the PCA, to reduce the 88-12/88L-DRB1 haplotype numbers as explanatory variables, we used the haplotype frequencies composed of the field-1 level alleles. This level reflects differences in immuno-responsiveness due to changes in the amino acid sequences of the peptide-binding region and T-cell recognition region of each DLA allele [33]. The inbreeding coefficient (Fis) and haplotype richness (Hr) were calculated by FSTAT Ver. 2.9.4 (available from https://www2.unil.ch/popgen/softwares/fstat.htm (accessed on 21 June 2021). In this case, the significant deviation of FIS from zero was also tested by FSTAT Ver. 2.9.4. Pearson product-moment correlation coefficient was calculated with R ver. 3.6.3 (available from https://www.r-project.org/ (accessed on 17 March 2020)) to evaluate differences in *DLA-DRB1* allele diversity in the same dog breeds from different countries, UK and Japan. A phylogenetic tree was constructed by the Neighbor Joining method and assessed using 10,000 bootstrap replicates after aligning the DLA sequences using the MEGA X software (available from https://www.megasoftware.net/ (accessed on 3 March 2020) [36]. A pairwise sequence similarity plot was displayed by a graphical user interface GenomeMatcher [37].

## 3. Results

### 3.1. Allele Number and Frequency of DLA-88, DLA-88L, DLA-12, and DLA-DRB1

**Figure 2:**
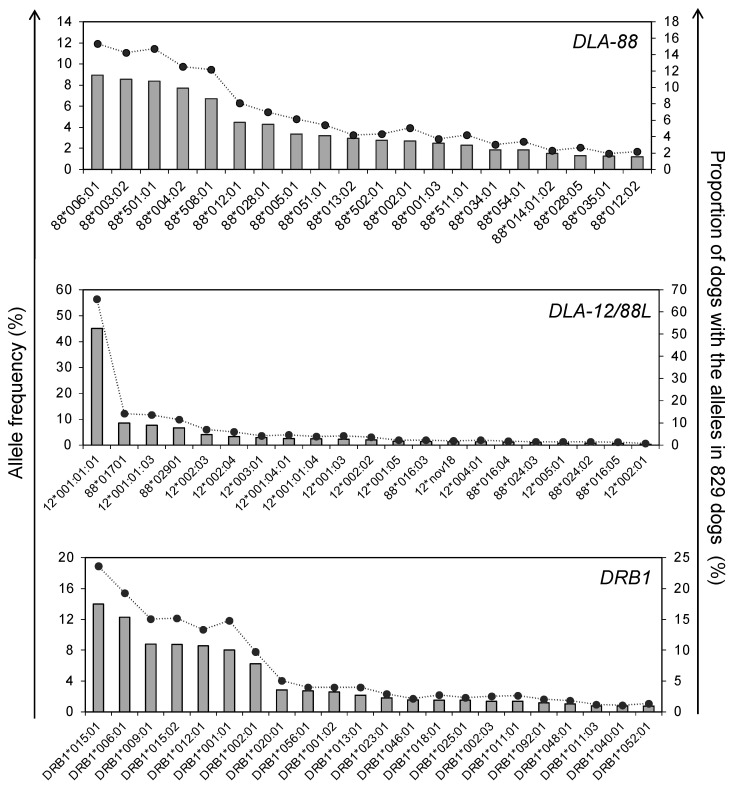
Top 20, 21 or 22 most frequent alleles of *DLA-88*, *DLA-12/88L* and *DLA-DRB1* loci detected for 829 dogs. The allele frequency and the proportion of dogs with each allele are described by a bar and line chart, respectively. The allele names are indicated below each graph.

Table 2 and Supplementary Table S2 show detailed information on the types, numbers, and frequencies of *DLA-88*, *DLA-88L*, *DLA-12,* and *DLA-DRB1* alleles identified in the 829 dogs. In total, 193 DLA alleles (89 in *DLA-88*, 18 in *DLA-88L*, 25 in *DLA-12*, and 61 in *DLA-DRB1*) were identified, and 17, 7, 5, and 6 were novel alleles of *DLA-88*, *DLA-88L*, *DLA-12*, and *DLA-DRB1*, respectively. Figure 2 shows frequencies of the 20 most frequent DLA alleles and the number of animals carrying the alleles in popular breeds in Japan. The highest frequent alleles in each DLA gene were DLA-88*006:01 (allele frequency: 8.9%), DLA-12*001:01:01 (45.1%), and DLA-DRB1*015:01 (14.0%). Although 43 *DLA-12/88L* alleles were identified, 65.7% (545 dogs) carried DLA-12*001:01:01. In the *DLA-DRB1* gene, DLA-DRB1*015 group alleles (DLA-DRB1*015:01, DLA-DRB1*015:02, DLA-DRB1*015:03, and DLA-DRB1*015:04) were the most common, and 37.7% had the alleles. This result showed a similar ratio (23.6%) to the previously published report [20].

### 3.2. Phylogenetic Relationships of the DLA-88, DLA-88L, and DLA-12 Alleles

**Figure 3:**
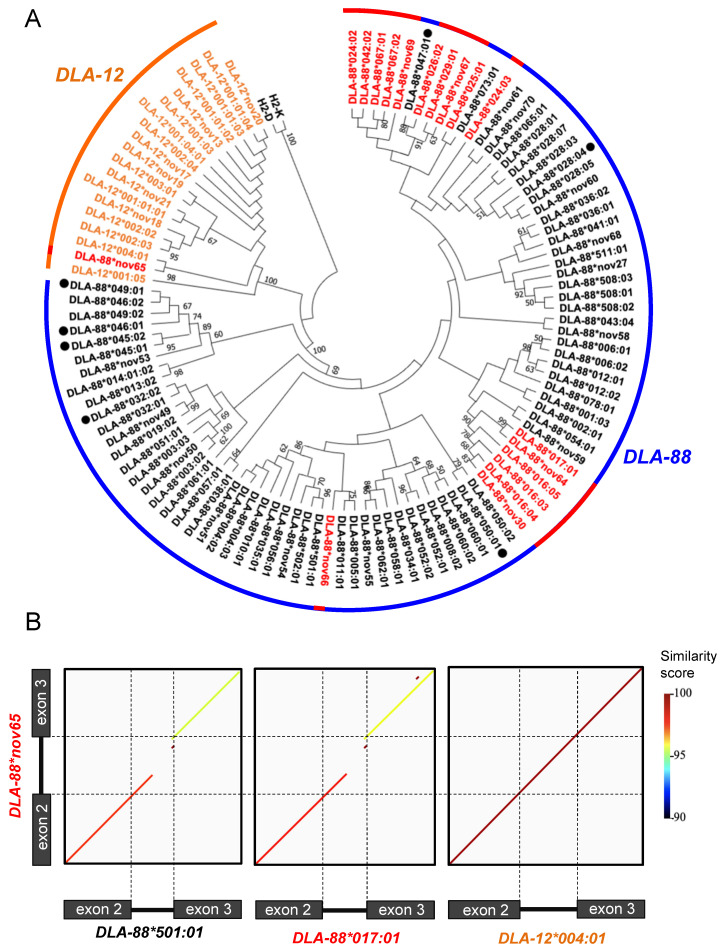
Phylogenetic relationship among the *DLA-88*, *DLA-12*, and *DLA-88L* allele sequences. (**A**) Neighbor-joining phylogenetic tree with exon 2–intron 2–exon 3 sequences of 109 alleles of *DLA-88*, *DLA-12*, and *DLA-88L*. The *DLA-12* and *DLA-88L* alleles are represented with orange and red characters, respectively. Alleles that were not detected from 829 dogs in our study sample are indicated with a black-filled circle. Nucleotide sequences of H-2D and H-2K are used as an outgroup of the tree. (**B**) Dot-match analysis between two alleles by GenomeMatcher. The sequence similarity between DLA-88*nov65 and DLA-88*501:01, DLA-88*017:01 or DLA-12*004:01 are displayed by lines. The lines are colored based on the similarity score calculated by the bl2seq program with parameter as follow: word size 10 and e-value threshold 0.1. When the similarity score was lower than 90, the line was not described at the region. Dashed lines indicate the boundary of exon and intron in each allele.

A phylogenetic tree of 109 different alleles was reconstructed using the *DLA-88*, *DLA-12*, and *DLA-88L* nucleotide sequences of exon 2–intron 2–exon 3 without indels (746 bp: alignment length). Ninety-nine of the 109 alleles were identified in the 426 dogs that we sequenced in this study. Another 10 *DLA-88* nucleotide sequences of exon 2–intron 2–exon 3 were obtained from the IPD-MHC and NCBI databases, and seven (DLA-88*028:04, DLA-88*032:02, DLA-88*045:02, DLA-88*046:01, DLA-88*047:01, DLA-88*049:01 and DLA-88*050:01) were not detected in our study. The phylogenetic tree clearly divided the *DLA-88* and *DLA-12* alleles into two lineages, and 16 of the 17 *DLA-88L* alleles were included in the *DLA-88* lineage (Figure 3A). The phylogenetic tree clearly divided the *DLA-88* and *DLA-12* alleles into two lineages, and 16 of the 17 *DLA-88L* alleles were included in the *DLA-88* lineage (Figure 3A). Of the 17 *DLA-88L* alleles, 14 were composed of two separate lineages containing two common alleles, DLA-88*017:01 and DLA-88*029:01 (Supplementary Table S2). In contrast, DLA-88*nov65, which was assigned as a *DLA-88L* allele, aligned with the *DLA-12* lineage, and its nucleotide sequence showed a high similarity of 99.87% to DLA-12*004:01 (Figure 3B). The intron 2 sequence of DLA-88*nov65 was highly different from DLA-88*501:01 and DLA-88*017:01, which grouped with the *DLA-88* and *DLA-88L* alleles, respect ively (Figure 3B).

### 3.3. Evaluation of DLA-DRB1 Polymorphisms between Same Dog Breeds in Japan and the United Kingdom

**Figure 4:**
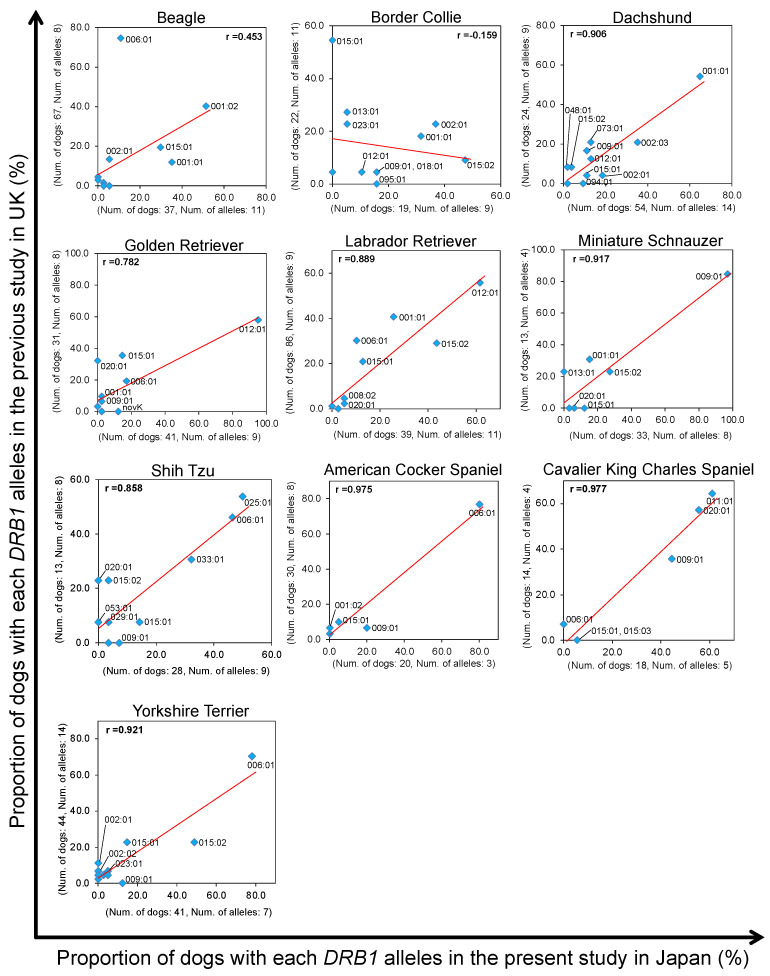
Comparisons of the proportion of dogs with each *DLA-DRB1* allele in 10 breeds between the present Japanese and the previous UK study. Scatter plots based on the proportion of dogs with the *DLA-DRB1* alleles in ten breeds are shown. The number of dogs analyzed and the number of observed alleles in the present Japanese and the previous UK study are described on the *x*- and *y*-axis in each plot, respectively. Dachshunds are summarized without classification by their size, such as miniature or kaninchen, and hair length, such as short or long, in both studies. An allele name is displayed alongside a marker when the proportion is above 5% in either the present or previous study. A red line in each plot represents a regression line. Also, a correlation coefficient (r) is indicated in each plot.

The migration or transportation of dog breeds between different geographic locations has been shown to have a detectable effect on breed structures with the generation of genetically differentiated sub-populations [38,39,40]. Therefore, to evaluate the genetic bias of the DLA polymorphisms between the same dog breeds in Japan and another country, we compared our *DLA-DRB1* genotyping data with the previously published *DLA-DRB1* polymorphism data in the United Kingdom (UK) [21]. We compared the proportion of the dogs with each of the different *DLA-DRB1* alleles in 10 breeds that had been analyzed in more than 12 dogs per breed in the present (Japan) and previous studies (UK). Of the 10 breeds in the UK and Japan, moderate to strong correlations with correlation coefficients ranging from 0.453 (Beagle) to 0.977 (Cavalier King Charles Spaniel), and a median value of 0.853 was confirmed in the nine breeds (Beagle, Golden Retriever, Labrador Retriever, Dachshund, Miniature Schnauzer, American Cocker Spaniel, Cavalier King Charles Spaniel, Shih Tzu, and Yorkshire Terrier) (Figure 4). The Beagles showed a moderate correlation coefficient, but a large difference was observed between the two countries in the proportion of individuals carrying DLA-DRB1*006:01 (74.6% in the UK vs. 10.8% in Japan). However, for Border Collies, whereas 7 out of 10 alleles were commonly observed in both countries, the *DLA-DRB1* allele frequency differed markedly, and no positive correlation was observed between the proportions (correlation coefficient r: −0.159) of alleles in each country.

### 3.4. Frequency of the 88-12/88L-DRB1 Haplotypes

To identify the two 88-12/88L-DRB1 haplotypes (88-12-DRB1 or 88-88L-DRB1) within the 829 dogs, we searched homozygous dogs with the three-loci, any two-loci (88-12/88L, 88-DRB1 and 12/88L-DRB1) and one-locus from our genotyping data. Firstly, 52 different sub-haplotypes of the 88-12/88L-DRB1 haplotypes were identified within 198 dogs (23.8%) that were homozygous at the three loci. Then, 54 sub-haplotypes of the 88-12/88L-DRB1 haplotypes were identified within 40 dogs and 163 dogs that were homozygous at two-loci (88-12/88L in 7 dogs, 88-DRB1 in 7 dogs, 12/88L-DRB1 in 16 dogs) and at one-locus (*DLA-88* in 3 dogs, *DLA-12/88L* in 111 dogs, and *DLA-DRB1* in 49 dogs), respectively. 

In total, 106 sub-haplotypes of the 88-12/88L-DRB1 haplotypes were identified. In addition, 84 sub-haplotypes of 88-12/88L-DRB1 were estimated from the haplotype estimation of the 428 remaining heterozygous dogs with reference to the 106 sub-haplotypes of the 88-12/88L-DRB1 haplotypes. Consequently, 190 88-12/88L-DRB1 sub-haplotypes in total were obtained from 803 dogs of 49 breeds (Supplementary Table S3). However, the remaining 26 dogs could not be assigned to the 88-12/88L-DRB1 haplotypes, because the combination of sub-haplotypes was not narrowed down to less than three loci. Of 190 88-12/88L-DRB1 sub-haplotypes, 131 were detected two or more times, while the other 59 sub-haplotypes were detected just once within heterozygous dogs (Supplementary Table S3B). The frequencies of 131 sub-haplotypes within the 88-12-DRB1 and the 88-88L-DRB1 haplotype structures were 79.4% and 20.6%, respectively (Table 3).

Of the 131 different 88-12/88L-DRB1 haplotypes, 29 were high-frequency haplotypes with a frequency of 1.0% or more (i.e., the detected number of the haplotype was ≥16) (Table 4). Of these 29 highly frequent haplotypes, four haplotypes (DLA-88*006:01–DLA-12*001:01–DRB1*056:01 (Haplotype(Hp)-ID 116), DLA-88*502:01–DLA-12*001:01–DRB1*001:02 (Hp-ID 6), DLA-88*001:03–DLA-12*001:01–DRB1*046:01 (Hp-ID 91), and DLA-88*511:01–DLA-12*001:03–DRB1*092:01:1 (Hp-ID 117) were observed in only one breed. The other 25 haplotypes were observed in two or more breeds, and 11 sub-haplotypes (Hp-IDs 2, 20, 22, 23, 37, 46, 51, 52, 69, 73, and 99) showed high haplotype frequencies of 70% or more in specific dog breeds (Table 4). Therefore, more than 50% of the high-frequency 88-12/88L-DRB1 haplotypes (15 of 29 haplotypes) were found in breeds with a large number of dogs tested. DLA-88*004:02–DLA-12*001:01–DRB1*006:01 (Hp-ID 12) was detected as the most frequent haplotype in two breeds, Pomeranian (haplotype frequency: 46.6%) and Yorkshire Terrier (Hp frequency: 31.7%) (Supplementary Table S3A). In addition, the DLA-88*003:02–DLA-88*017:01–DRB1*009:01 (Hp-ID 31), and DLA-88*501:01–DLA-12*001:01:01–DRB1*001:01 (Hp-ID 8) were commonly observed in multiple breeds with relatively similar frequencies (Supplementary Table S3A).

### 3.5. Comparison of Genetic Diversity between Dog Breeds by Haplotype Numbers and Heterozygosity

**Figure 5:**
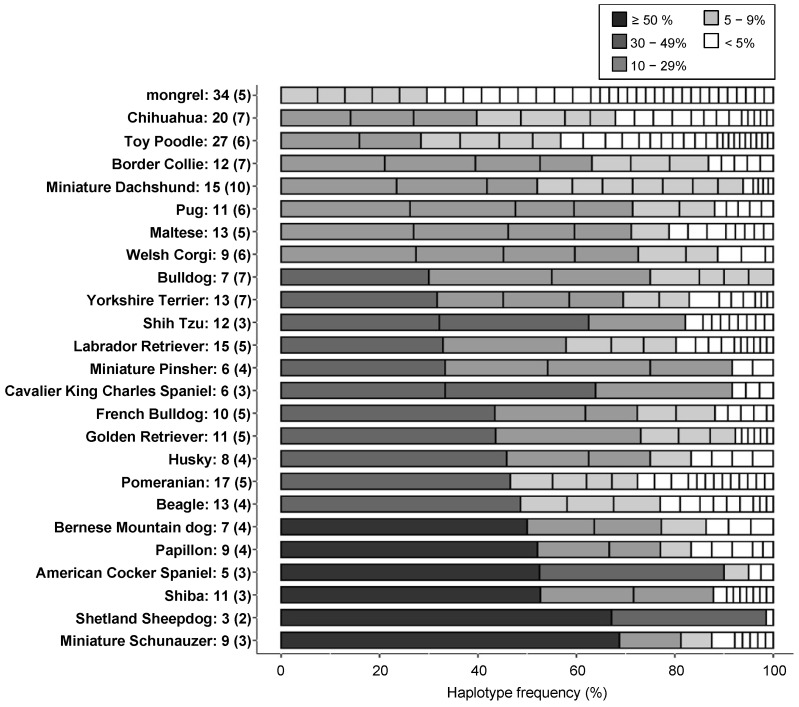
Percentage distribution of the 88-12/88L-DRB1 haplotypes in 24 breeds and mongrels. The cumulative bar chart is based on the frequency of all the 88-12/88L-DRB1 haplotypes detected from each breed listed in Table 5. A numeral following the breed’s name indicates the number of different haplotypes detected in each breed. Parenthesis indicates the number of different 88-12/88L-DRB1 haplotypes with a frequency of 5% or higher. The breeds are sorted in ascending order by the percentage frequency of the most frequent haplotype in each breed. For example, the graph of Miniature Schnauzer at the bottom of the figure represents a total of nine sub-haplotypes of 88-12/88L-DRB1 haplotypes with a frequency of 5% or higher for three of them at >50%, 10–29%, and 5–9%, respectively.

We investigated the genetic diversity of the 88-12/88L-DRB1 haplotypes using a total of 725 dogs within 24 different dog breeds (analyzed using ≥ 10 dogs/breed) and mongrels (mixed breeds). The number of different haplotypes in each breed ranged from three Shetland Sheepdogs to 27 Toy Poodles (Table 5), and up to 34 different haplotypes among the mongrels (Figure 2). Six dog breeds (Miniature Schnauzer, Shetland Sheepdog, Shiba, American Cocker Spaniel, Papillon, and Bernese Mountain Dog) had one particular 88-12/88L-DRB1 sub-haplotype at a frequency of greater than 50% (Figure 5). Only two or three haplotypes represented more than 80% of all the haplotypes in seven breeds (Miniature Schnauzer, Shetland Sheepdog, Shiba, American Cocker Spaniel, Golden Retriever, Miniature Pinscher, and Shih Tzu). In contrast, more than 20 different haplotypes were detected in Chihuahua and Toy Poodle, and each haplotype frequency was distributed similarly (Figure 5).

We calculated the genetic diversity indices, such as observed heterozygosity (Ho), expected heterozygosity (He), inbreeding coefficient (Fis), and haplotype richness (Hr) to evaluate the 88-12/88L-DRB1 diversity in each breed (Table 5). The mean Ho value in the 24 dog breeds was 0.736. The Ho values deviated significantly from HWE in 8 breeds (Cavalier King Charles Spaniel, Golden Retriever, Labrador Retriever, American Cocker Spaniel, Shiba, Papillon, Shih Tzu, and Beagle), and the Ho values were significantly lower than He values in five breeds (American Cocker Spaniel, Shiba, Papillon, Shih Tzu, and Beagle). The Hr values in the 24 breeds ranged from 2.13 in Shetland Sheepdog to 7.79 in Toy Poodle.

### 3.6. Characteristics of Genetic Relationship of the 88-12/88L-DRB1 Haplotypes by Principal Component Analysis

**Figure 6:**
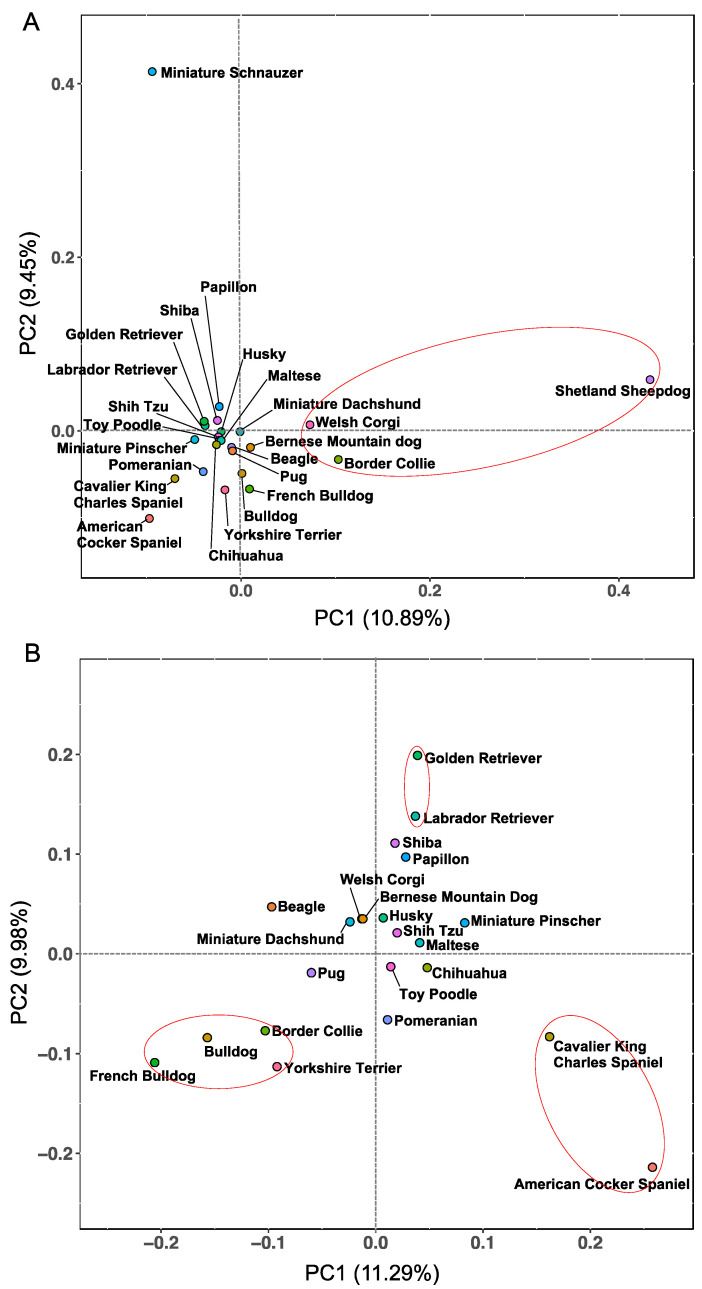
Principal component analysis based on 88-12/88L-DRB1 haplotype frequencies among 24 breeds. The analysis based on the frequency of three-locus DLA haplotypes in each allele at field-1 level among (**A**) 24 breeds listed in Table 5 and (**B**) 22 breeds except for Shetland Sheepdog and Miniature Schnauzer from (**A**) are represented. The breeds that shared a three-loci haplotype with relatively high frequency (See Section 3.6. in detail) are grouped with a red circle. The contribution ratio of the first (PC1) and second component (PC2) are described on the *x*- and *y*-axis in parentheses, respectively.

To evaluate genetic relationship of the 88-12/88L-DRB1 haplotypes among different dog breeds, PCA was performed using the 24 breeds listed in Table 5. Of the 24 breeds plotted by PCA, 22 breeds were distributed closely around the centroid of the quadrants as if they were almost one population. The Shetland Sheepdog and Miniature Schnauzer breeds diverged markedly from the other 22 breeds. (Figure 6A and Supplementary Table S3A). The positions of the Shetland Sheepdog and Miniature Schnauzer breeds within the matrix appear to have reflected the presence of their dominant haplotypes, 88*003–88*017–DRB1*002 (Hp-ID 20) in Shetland Sheepdog (Hp frequency: 67.1%) and 88*013–12*003–DRB1*009 (Hp-ID 23) in Miniature Schnauzer (68.8%). Since the 88*003–88*017–DRB1*002 (Hp-ID 20) also were commonly observed among Welsh Corgi and Border Collie, these two breeds were located slightly outside the large group of the other breeds and closer to Shetland Sheepdog. Removing the Shetland Sheepdog and Miniature Schnauzer outliers from the PCA analysis changed the genetic relationship slightly between some of the 22 breeds on the basis of the 88-12/88L-DRB1 haplotype frequencies (Figure 6B). For example, the French Bulldog, Bulldog, Border Collie, and Yorkshire Terrier share the 88*028–88*029–DRB1*015 (Hp-IDs 24 and 25) at relatively high frequencies (13.4% to 43.4%), and these four breeds grouped more closely together and at some distance from the other breeds. Similarly, the Golden retriever and Labrador retriever, sharing the 88*508–12*001–DRB1*012 (Hp-ID 21), and American Cocker Spaniel and Cavalier King Charles Spaniel, sharing the 88*003–88*017–DRB1*009 (Hp-ID 31) separated further from each other and at a greater distance from the centroid (0, 0) of the PCA plot (Figure 6B).

### 3.7. Number of Potential Recipients for 88-12/88L-DRB1-Matched Transplantation, Assuming Homozygous-Derived Somatic Stem Cells as Donors

**Figure 7:**
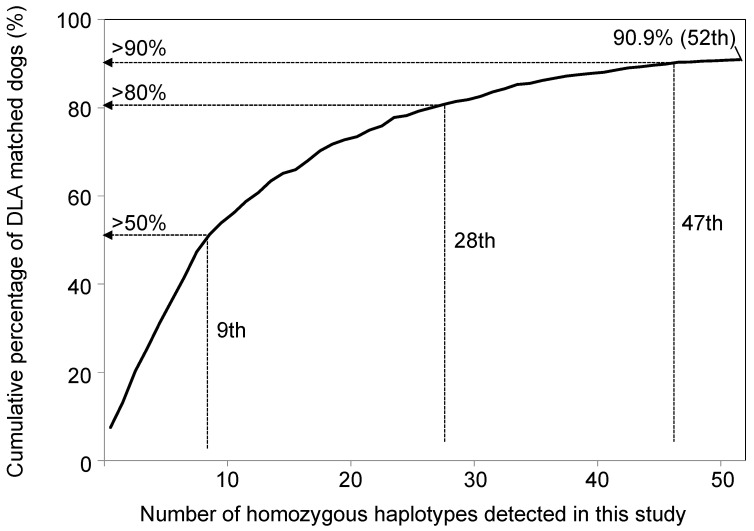
The cumulative percentage of dogs matched for homozygous 88-12/88L-DRB1 haplotypes detected in this study. The horizontal and vertical axes indicate 52 types of 88-12/88L-DRB1 homozygous haplotype numbers, which are sorted in descending order of the haplotype frequency, and the number of dogs, respectively. The cumulative proportion of dogs was calculated using the 803 dogs with identified haplotypes.

Assuming that somatic stem cells could be established from the 52 types of homozygotes of the 88-12/88L-DRB1 haplotypes and that these cells could be used as donors for 88-12/88L-DRB1-matched transplantations, we statistically modeled the number of dogs that might be recipients from the 829 individuals analyzed in this study (Figure 7). From our statistical simulation, if donor cells are established from 9, 28, and 52 types of high frequency 88-12/88L-DRB1 homozygotes, 411 (51.2%), 650 (80.7%), and 733 (90.9%) dogs might be considered eligible for 88-12/88L-DRB1-matched transplantation as recipients. Furthermore, in 17 of 24 dog breeds (70.8%) listed in Table 5, 50% or more of these dogs might benefit from the 88-12/88L-DRB1-matched transplantation by using donor dogs with the most frequent 88-12/88L-DRB1 haplotypes in each breed (Table 6). Additionally, homozygotes of all haplotypes listed in Table 6, except 88*006:01–DLA-12*001:01–DRB1*015:01 (Hp-ID 37), were detected in the present study (Supplementary Table S3A).

## 4. Discussion

In this study, we genotyped the *DLA-88*, *DLA-12/88L*, and *DLA-DRB1* loci by Sanger sequencing using 829 dogs of 59 breeds and identified 89, 43, and 61 alleles, respectively. We also developed a two-stage PCR method for the polymorphism analysis of the *DLA-88*, *DLA-88L*, and *DLA-12* genes by separating *DLA-88* and *DLA-12/88L* with the 1st PCR and *DLA-12* and *DLA-88L* with the 2nd PCR (Figure 1B). This polymorphism analysis by PCR and sequencing clearly distinguished the DLA-88L allele from the *DLA-88* allele, which was difficult with conventional RNA-based methods [15,18]. In fact, the previously reported DLA-88*042:02 [17] belongs to *DLA-88L* rather than *DLA-88*, and this allele along with DLA-88*008:02 and DLA-DRB1*004:01 constituted the 88-88L-DRB1 haplotype in the Maltese breed (Supplementary Table S2A). Therefore, this simpler and more accurate two-stage PCR method is an important tool to use for a better understanding of the DLA loci and haplotype differences and for evaluating various immune responses in dogs.

Overall, we identified 29 novel *DLA-88*, *DLA-88L*, and *DLA-DRB1* alleles in this study. Of them, DLA-88*nov65 was newly detected as a *DLA-88L* allele that showed a different phylogenetic relationship from other DLA-88L alleles, and was highly similar to DLA-12*004:01 of the *DLA-12* lineage (Figure 3). Interestingly, our previous study showed that DLA-12*004:01 was generated by a gene conversion event within the exon 2 region between the *DLA-12* and *DLA-88* alleles [30]. Therefore, the DLA-88*nov65 also might have been generated by gene conversion between the *DLA-88* and *DLA-12* alleles, similar to DLA-12*004:01. There may be many other unidentified DLA alleles generated by such gene conversions events.

In contrast to the DLA-I genes, the polymorphisms and diversity analyses of the DLA-II genes (*DLA-DRB–DLA-DQA–DLA-DQB*) have been performed previously in many different dog breeds [21,22,41,42]. Although the Japanese native species of Shiba has not been well analyzed previously, our current analysis showed that DLA-DRB1*056:01 (allele frequency: 55.4%) was the most frequent allele, followed by DLA-DRB1*092:01:1 (18.9%), and DLA-DRB1*011:03 (16.2%) (Supplementary Table S3A). These *DLA-DRB1* alleles were detected only in Shiba, and therefore their detection is extremely rare even in past polymorphism analyses of the DLA-II genes, including other dog breeds of Asian origin [21,42]. The DLA polymorphism information on Japanese native breeds is extremely limited [42,43]. In this study, although we analyzed Japanese native species Shiba, Akita, Japanese Spitz, Chin, and Shikoku, the number of animals analyzed was less than 10 animals except for the 37 in the Shiba breed. Therefore, more DLA allele information is necessary for Japanese native species as well as for dog breeds that have not yet been analyzed.

Recent genomic analysis of the remains of extinct Japanese wolves (*Canis lupus hodophilax*) showed phylogenetically that after the Japanese wolf and modern dog ancestry had diverged from grey wolf lineages, gene flow occurred from the ancestor of Japanese wolves into the ancestor of Japanese dogs, including Shiba, and this flow likely continued and contributed to differentiate between the lineage of Japanese dogs and West Eurasian dogs [44]. Interestingly, DLA-DRB1*056:01 of Shiba was detected in Finnish and Russian wolves with frequencies of 4.0% and 2.9%, respectively [45], and Shiba DLA-DRB1*092:01:1 was detected in Canadian and Croatian wolves with frequencies of 6.0% and 11.0%, respectively [46,47]. These results indicated that Shiba might be a unique breed that shared some of its genomic sequences, including the DLA genomic region, with its ancestor in a different way than those of the European modern dog breeds.

High homozygosity of the DLA haplotypes generally implies a loss of DLA genetic diversity. The Ho values showed significantly lower values than the He values in 5 dog breeds, American Cocker Spaniel, Shiba, Papillon, Shih Tzu, and Beagle (Table 5). This suggests a high level of inbreeding in these five breeds. The high Fis values also observed in Shih Tzu (0.209) and Papillon (0.171) strongly suggest that the DLA diversity in these breeds of our population sample is decreasing by inbreeding (Table 5). Moreover, Shetland Sheepdog showed an extremely low Ho value of 0.314 (Table 5). The loss of genetic diversity due to high homozygosity might increase the deleterious genetic variation in pure-breed dogs [48]. Also, high homozygosity of the DLA region due to both inbreeding and genetic bottlenecks by selective artificial breeding was associated with the development of autoimmune diseases in Italian Greyhounds [49]. In contrast, MHC heterogeneity of the Sea lion in wild populations appears advantageous to protect against infectious diseases [50], whereas the pregnancy rate in horses was reported to be decreased by sharing common MHC types between males and females [51]. Therefore, loss of the DLA diversity may affect biological fitness as homozygosity progresses.

The homozygous rate of *DLA-DRB1–DLA-DQA1–DLA-DQB1* haplotypes was 35% in a previous study [22]. These three DLA-II genes are located together within 100 kb, while *DLA-88* is located far from *DLA-DRB1* by over 1.0 Mb [13], resulting in a much stronger linkage disequilibrium (LD) within the *DLA-DRB1–DLA-DQA1–DLA-DQB1* haplotype than the 88-12/88L-DRB1 haplotype. Therefore, the lower 88-12/88L-DRB1 homozygous rate (23.8%) in this study than that of *DLA-DRB1–DLA-DQA1–DLA-DQB1* haplotype in the previous study [22] might be associated with LD and rates of recombination between different DLA gene loci. The detection of 52 types of homozygotes for the 88-12/88L-DRB1 haplotypes suggests that 90.9% of the dogs analyzed in our study would have a successful 88-12/88L-DRB1-matched transplantation if their somatic stem cells were used in such a procedure. In comparison, if induced pluripotent stem cells (iPSCs) were established from homozygotes of 30 and 50 types of HLA haplotypes (*HLA-A–HLA-B–HLA-DRB1*) that are frequently observed in Japanese, 82.2% and 90.7% of Japanese would benefit from HLA-matched iPSC transplantation [52]. However, the HLA homozygosity rate for humans is relatively low at 0.5 to 1.5% for the *HLA-A–HLA-B–HLA-DRB1* haplotype [53,54,55]. Therefore, the HLA of 15,000 and 24,000 individuals would need to be genotyped to identify these 30 and 50 HLA homozygotes, respectively [52]. In this regard, the MHC homozygotes, preferred donors for somatic stem cell sources, would be much easier to detect in dogs than in humans due to their higher rate of MHC homozygosity. Moreover, our new data on the frequency of DLA haplotypes in various dog breeds could help in the implementation of somatic stem cell transplantation along with a recent development of clinical-grade canine iPSCs derivation [56,57] and assist with the high expectations for regenerative medicine in the veterinary field [58].

In the PCA using the 88-12/88L-DRB1 haplotype frequencies, the values of the first principal component (PC1) and the second principal component (PC2) were extremely low at around 10% in both analyses, but with relatively strong diversity between the DLA haplotypes among the 24 dog breeds, which are popular in Japan (Figure 6). The Hr values of 88-12/88L-DRB1 were less than five in 11 breeds, suggesting that there were relatively few breeds with many different types of 88-12/88L-DRB1 haplotypes (Table 5). Moreover, haplotype frequency bias was confirmed for six breeds, with some haplotype frequencies at 50% or more within each breed (Figure 5). These results showed that the DLA diversity is highly conserved within most breeds, but divergent between almost all breeds, as similarly observed in a previous study on the DLA-II haplotype diversity [20,22]. Therefore, the DLA allele and DLA haplotype frequencies appear to change largely depending on the breeds. Of the 829 dogs of 59 breeds analyzed in this study, 68.5% (568 of 829 dogs) were from the top 20 most popular breeds registered in Japan (Table 1). In contrast, only a few dogs were analyzed from breeds that are listed in the top ten most popular breeds in the USA (American Kennel Club; https://www.akc.org/ (accessed on 28 November 2022)), such as German Shepherd and Rottweiler, which were less popular breeds in Japan. The genetic closeness of the DLA haplotypes among different breeds can be evaluated more accurately by enhancing the DLA genotyping data of breeds from which only a small number of dogs were analyzed in our present study. We showed that there are differences in the distribution of *DLA-DRB1* alleles within the same breeds, such as Border Collie and Beagle, particularly if they are located in different countries, such as Japan and the UK (Figure 4). Since differences in the DLA diversity within a single breed between different countries have also been reported in some other breeds [49,59,60], geographical location can affect DLA diversity resulting in differences in the susceptibility for various diseases even in a single breed between different countries. From such a discussion, further DLA polymorphisms analysis for various breeds in different countries is warranted to better comprehend the intriguing features of DLA diversity.

A limitation of our study concerning DLA haplotypes containing both DLA-I and DLA-II genes was not to include *DLA-DQA1* and *DLA-DQB1* polymorphisms that might be linked to the 88-12/88L-DRB1 haplotypes. This was beyond the scope of our present study. The three genes of *DLA-DRB1*, *DLA-DQA1*, and *DLA-DQB1* often show strong linkage disequilibrium [22], but novel *DLA-DRB1–DLA-DQA1–DLA-DQB1* haplotypes might be generated by the recombination between the DLA-DR and DLA-DQ genes. Although the mismatch of HLA-DQ polymorphisms in organ transplantation is associated with the production of de novo donor-specific antibodies (DSA) against the HLA-DQ molecules and contributes to poor graft outcomes [61,62], such studies are lacking in dogs. Therefore, in regard to transplantations in dogs, further studies are necessary to genotype *DLA-DQA1* and *DLA-DQB1* and consolidate the extended haplotypes that were identified in our study, including all DLA-I and DLA-II genes, to select the most suitable organ donors in future.

Several specific DLA-II alleles and haplotypes have been reported so far to associate with various diseases in different breeds. For example, DLA-DRB1*010:01:1 or the DLA-DRB1*010:01:1–DLA-DQA1*002:01–DLA-DQB1*015:01 haplotype is associated significantly with the risk of necrotizing meningoencephalitis in Pug [63]. DLA-DRB1*094:01 is associated significantly with acquired retinal degradation syndrome in Dachshunds [64]. These susceptible *DLA-DRB1* alleles were also detected in the Pugs and Dachshunds in our study (Supplementary Table S3A). However, the association between the DLA alleles and diseases is unknown because we did not survey the medical history of the individuals used in this study. Also, we have not evaluated the association between decreased heterozygosity of the DLA haplotype and decreased biological fitness. Considering these limitations of the present study, we would like to elucidate the association between DLA polymorphisms and diseases and fertility in the future by investigating the disease history in each of the major breeds in our study population.

## 5. Conclusions

The genetic diversity of DLA haplotypes varied remarkably between breeds but was relatively conserved within the breed in our large-scale polymorphism analysis. The genetic characteristics of the high DLA homozygosity rate and poor DLA diversity within the same breed are useful for transplantation therapy, but they also may affect biological fitness negatively as homozygosity progresses. This DLA polymorphism information might be useful in future studies for the realization of canine transplantation medicine and elucidation of the pathology of various diseases and for the development of DLA-haplotype-based veterinary medicine.

## Figures and Tables

**Table 1:** Number of dogs in each of the 59 breeds and mongrels analyzed in this study.

Breed	Number of Dogs	^a^ Rank of the Number of Dogs Registered in Japan	Breed	Number of Dogs	^a^ Rank of the Number of Dogs Registered in Japan
Miniature Dachshund	49	3	Kaninchen Dachshund	5	
Toy Poodle	44	1	Bichon frize	4	16
Golden Retriever	41	11	Cairn Terrier	4	
Yorkshire Terrier	41	8	Japanese Spitz	4	28
Beagle	39	24	Pekingese	4	19
Chihuahua	39	2	Standard Poodle	4	
Labrador Retriever	39	13	Chin	3	
French Bulldog	38	6	Chinese Crested Dog	3	
Shiba	37	9	Doberman	3	
Shetland Sheepdog	35	23	Great Pyrenees	3	
Miniature Schnauzer	33	5	Kooikerhondje	3	
Welsh Corgi	33	14	Saint Bernard	3	
Pomeranian	29	4	Dalmatian	2	
Shih Tzu	28	10	Norfolk Terrier	2	
Maltese	26	7	Weimaraner	2	
Papillon	24	15	Basenji	1	
Pug	21	12	Brussels Griffon	1	
American Cocker Spaniel	20	26	Rough Collie	1	
Border Collie	19	18	Irish Setter	1	
Cavalier King Charles Spaniel	18	22	Lakeland Terrier	1	
Husky	12	27	Leonberger	1	
Miniature Pinsher	12	21	Miniature Bull Terrier	1	
Bernese Mountain Dog	11	29	Rottweiler	1	
Bulldog	10	30	Saluki	1	
Akita	9		Shikoku	1	
Boston Terrier	6	25	Staffordshire Bull Terrier	1	
Italian Grey Hound	6	20	Toy Manchester Terrier	1	
Jack Russell Terrier	6	17	Wippet	1	
English Cocker Spaniel	5		mongrel	27	
Flat-Coated Retriever	5				
German Shepherd	5		Total	829	

^a^ The data of a rank (top 30 most popular) of the number of dogs for each breed in Japan registered in 2022 is available from the Japan kennel club website (https://www.jkc.or.jp/ (accessed on 1 February 2023)).

**Table 2:** Summary of the DLA alleles detected from 829 dogs of 59 breeds.

Locus	*DLA-88*	*DLA-12/88L*		*DLA-DRB1*
		*DLA-12*	*DLA-88L*	
Number of Nucleotide Sequences Detected in This Study	89	25	18	61
Previously published sequences	72	20	11	55
Novel sequences	17 (8)	5 (2)	7 (3)	6 (4)
^a^ Number of nucleotide sequences not detected in this study	56	0	-	108
Number of unique amino acid sequences	88	21	18	61

The number in the parenthesis of “Novel sequences” represents the number of allele sequences observed from two or more individuals in this study. The detailed information about all alleles detected in this study is described in Supplementary Table S2. ^a^ The numbers in the row indicate the number of registered sequences in the IPD-MHC database that were not detected in this study.

**Table 3:** Summary of the three-locus haplotypes assigned to 803 dogs of 49 breeds and mongrels.

Haplotype	Haplotypes Detected Two or More Times		Number of Other Single Haplotypes ^a^	Frequency (%)	Homozygote	
	Number of Sub-Haplotypes	Number of Haplotypes			Number of Sub-Haplotypes	Number of Dogs
*DLA-88–DLA-12/88L–DRB1*	131	1547	59	-	52	198
two haplotype structures						
*DLA-88–DLA-12–DRB1*	101	1228	36	79.4	42	152
*DLA-88–DLA-88L–DRB1*	30	319	23	20.6	10	46

^a^ The number represents the haplotypes that are observed just once from heterozygous dogs.

**Table 4:** The 29 most frequent 88-12/88L-DRB1 haplotypes found in this study.

Haplotype ID. ^a^	DLA-88*	*DLA-12/88L*		DLA-DRB1*	Number of Haplotypes	Haplotypefrequency (%)	Number of Dogs with the Haplotypes	Number of Homozygous Dogs	Number of Breeds with the Haplotype Except for Mongrels ^c^
		DLA-12*	*DLA-88L* ^b^ (DLA-88*)						
12	*004:02*	*001:01:01*	-	*006:01*	68	4.23	60	8	10
20	*003:02*	-	*017:01*	*002:01*	64	3.99	45	19	6 (Shetland Sheepdog (73.4%))
31	*003:02*	-	*017:01*	*009:01*	64	3.99	59	5	11
25	*028:01*	-	*029:01*	*015:02*	62	3.86	52	10	8
8	*501:01*	*001:01:01*	-	*001:01*	53	3.30	47	6	8
21	*508:01*	*001:01:03*	-	*012:01*	50	3.11	42	8	3
7	*012:01*	*001:01:01*	-	*015:01*	48	2.99	42	6	9
23	*013:02*	*003:01*	-	*009:01*	46	2.86	33	13	2 (Miniature Schunauzer (95.7%))
116	*006:01*	*001:01:01*	-	*056:01*	41	2.55	31	10	1 (Shiba (95.1%))
73	*051:01*	*001:01:01*	-	*012:01*	40	2.49	33	7	2 (Golden Retriever (85.0%))
6	*502:01*	*001:01:01*	-	*001:02*	38	2.37	28	10	1 (Beagle (94.7%))
63	*004:02*	*001:01:01*	-	*015:01*	34	2.12	28	6	14
18	*005:01*	*002:04*	-	*020:01*	32	1.99	28	4	9
17	*501:01*	*001:01:01*	-	*006:01*	31	1.93	27	4	8
2	*006:01*	*001:01:01*	-	*006:01*	30	1.87	24	6	8 (AmericanCocker Spaniel (70.0%))
51	*034:01*	*002:03*	-	*023:01*	29	1.81	23	6	5 (Shetland Sheepdog (75.9%))
91	*001:03*	*001:01:01*	-	*046:01*	26	1.62	18	8	1 (Papillon (96.2%))
46	*014:01:02*	*001:05*	-	*025:01*	25	1.56	19	6	4 (Shih Tzu (72.0%))
71	*002:01*	*001:01:01*	-	*011:01*	23	1.43	22	1	3
52	*006:01*	*001:01:01*	-	*002:03*	23	1.43	21	2	2 (Dachshund (78.3%))
66	*006:01*	*001:01:01*	-	*001:01*	22	1.37	22	0	6
10	*508:01*	*001:01:03*	-	*002:01*	21	1.31	21	0	7
22	*511:01*	*001:03*	-	*015:02*	21	1.31	19	2	3 (Laborador Retriever (90.5%))
99	*035:01*	*nov18*	-	*006:01*	20	1.25	15	5	2 (Shih Tzu (85.0%))
69	*054:01*	*002:02*	-	*012:01*	20	1.25	17	3	2 (Welsh Corgi (85.0%))
37	*006:01*	*001:01:01*	-	*015:01*	18	1.12	17	1	3 (Toy Poodle (77.8%))
48	*028:05*	-	*029:01*	*015:02*	18	1.12	18	0	6
33	*501:01*	*001:01:01*	-	*012:01*	16	1.00	16	0	5
117	*511:01*	*001:03*	-	*092:01:1*	16	1.00	15	1	1 (Shiba (87.5%))

^a^ Haplotype ID. is identical to the number defined in Supplementary Table S3A. ^b^ All *DLA-88L* alleles as well as *DLA-88* alleles are named with “DLA-88*” as a prefix. ^c^ The predominant breed in each haplotype, accounting for >70% of the detected number of haplotypes, is described in parenthesis, followed by the percentage of dogs with the haplotype in each of the predominant breeds.

**Table 5:** Genetic diversity indices of 25 breeds and mongrels based on three loci (88-12/88L-DRB1) haplotypes.

Breed	Number of Dogs ^a^	Number of Estimated Haplotypes	Number of Homozygous Dogs	Ho	He	HWE Test	Fis	Hr ^b^
Shetland Sheepdog	35	3	24 (68.6%)	0.314	0.450	-	0.315	2.13
American Cocker Spaniel	20	5	9 (45.0%)	0.550	0.580	*p* < 0.05	0.077	2.94
Miniature Schunauzer	32 (1)	9	12 (37.5%)	0.625	0.510	-	−0.21	3.62
Cavalier King Charles Spaniel	18	6	5 (27.8%)	0.722	0.716	*p* < 0.001	0.020	3.79
Shiba	37	11	14 (37.8%)	0.622	0.658	*p* < 0.001	0.069	3.95
Golden Retriever	39 (2)	11	11 (28.2%)	0.718	0.713	*p* < 0.001	0.006	4.26
Papillon	24	9	10 (41.7%)	0.583	0.687	*p* < 0.05	0.171	4.60
Shih Tzu	28	12	10 (35.7%)	0.643	0.762	*p* < 0.001	0.209	4.64
Miniature Pinsher	12	6	2 (16.7%)	0.833	0.771	-	−0.038	4.64
Bernese Mountain Dog	11	7	3 (27.3%)	0.727	0.698	-	0.006	4.79
French Bulldog	38	10	12 (31.6%)	0.684	0.751	-	0.102	4.87
Husky	12	8	5 (41.7%)	0.583	0.733	-	0.245	5.06
Beagle	37 (2)	13	11 (29.7%)	0.703	0.730	*p* < 0.05	0.051	5.10
Bulldog	10	7	2 (20.0%)	0.800	0.790	-	0.040	5.20
Labrador Retriever	38 (1)	15	7 (18.4%)	0.821	0.807	*p* < 0.001	0.003	5.50
Welsh Corgi	31 (2)	9	5 (16.1%)	0.848	0.837	-	0.015	5.62
Pomeranian	29	17	6 (20.7%)	0.793	0.757	-	−0.03	5.64
Yorkshire Terrier	41	13	3 (7.3%)	0.927	0.836	-	−0.096	5.85
Pug	21	11	4 (19.0%)	0.810	0.840	-	0.061	5.87
Maltese	26	13	5 (19.2%)	0.808	0.847	-	0.066	6.02
Miniature Dachshund	49	15	5 (10.2%)	0.898	0.874	-	−0.018	6.40
Border Collie	19	12	4 (21.1%)	0.789	0.871	-	0.121	6.52
Chihuahua	39	20	2 (5.1%)	0.949	0.917	-	−0.022	7.44
Toy Poodle	44	27	4 (9.1%)	0.909	0.927	-	0.031	7.79
Mongrel	27	34	1 (3.7%)	0.963	0.962	-	0.018	9.16

Ho: observed heterozygosity, He: expected heterozygosity, Fis: inbreeding coefficient, Hr: haplotype richness. ^a^ A number outside and inside the parenthesis indicates the number of dogs whose haplotype could be estimated and whose haplotype could not be estimated in each breed, respectively. ^b^ This table is sorted by the ascending order of the number of Hr. “-” in the column of the HWE test indicates no significant differences.

**Table 6:** The percentage of the dogs with the most frequent haplotype within each breed.

Breed	Haplotype ID. ^a^	Most Frequent Haplotype in Each Breed				The Number of Dogs with the Haplotype within the Breed (%)
		DLA-88*	*DLA-12/88L*		DLA-DRB1*	
			DLA-12*	*DLA-88L* ^b^ (DLA-88*)		
Miniature Schunauzer	23	*013:02*	*003:01*	-	*009:01*	32/33 (97.0)
Shetland Sheepdog	20	*003:02*	-	*017:01*	*002:01*	29/35 (82.9)
American Cocker Spaniel	31	*003:02*	-	*017:01*	*009:01*	16/20 (80.0)
Shiba	116	*006:01*	*001:01:01*	-	*056:01*	29/37 (78.4)
Pomeranian	12	*004:02*	*001:01:01*	-	*006:01*	22/29 (75.9)
Bernese Mountain Dog	17	*501:01*	*001:01:01*	-	*006:01*	8/11 (72.7)
Papillon	91	*001:03*	*001:01:01*	-	*046:01*	17/24 (70.8)
Beagle	6	*502:01*	*001:01:01*	-	*001:02*	26/39 (66.7)
French Bulldog	25	*028:01*	-	*029:01*	*015:02*	25/38 (65.8)
Golden Retriever	33	*501:01*	*001:01:01*	-	*012:01*	26/41 (63.4)
Cavalier King Charles Spaniel	71	*002:01*	*001:01:01*	-	*011:01*	11/18 (61.1)
Yorkshire Terrier	12	*004:02*	*001:01:01*	-	*006:01*	24/41 (58.5)
Husky	77	*060:02*	*001:01:04*	-	*040:01*	7/12 (58.3)
Labrador Retriever	21	*508:01*	*001:01:03*	-	*012:01*	21/39 (53.8)
Bulldog	25	*028:01*	-	*029:01*	*015:02*	5/10 (50.0)
Miniature Pinscher	63	*004:02*	*001:01:01*	-	*015:01*	6/12 (50.0)
ShihTzu	46	*014:01:02*	*001:05*	-	*025:01*	14/28 (50.0)
Welsh Corgi	69	*054:01*	*002:02*	-	*012:01*	15/33 (45.5)
Maltese	7	*012:01*	*001:01:01*	-	*015:01*	11/26 (42.3)
Miniature Dachshund	8	*501:01*	*001:01:01*	-	*001:01*	20/49 (40.8)
Pug	110	*058:01*	-	*024:03*	*010:011*	8/21 (38.1)
Border Collie	24	*028:03*	-	*029:01*	*015:02*	6/19 (31.6)
Toy Poodle	37	*006:01*	*001:01:01*	-	*015:01*	13/44 (29.5)
Chihuahua	30	*508:01*	*001:01:03*	-	*015:01*	10/39 (25.6)

^a^ Haplotype ID. is identical to the number defined in Supplementary Table S3A. ^b^ The prefix of official name for *DLA-88L* alleles is “DLA-88*”.

## Data Availability

Not applicable.

## Supplementary Materials

The following supporting information can be downloaded at: https://www.mdpi.com/article/10.3390/cells12050809/s1, Supplementary Table S1: Information on primers using this study; Supplementary Table S2: Detailed information on each allele detected in this study; Supplementary Table S3: Summary of estimated the 88-12/88L-DRB1 haplotypes of 803 dogs with 49 breeds and mongrels.

## Nomenclature

Haplotype(s)	A set of closely linked alleles on a chromosome carrying the three-gene loci combination of 88-12-DRB1 or 88-88L-DRB1 and represented by 88-12/88L-DRB1 where 12/88L could be either 12 or 88L.
Sub-haplotype(s)	A single locus (88, 12, 88L, 12/88L, DRB1) or two-locus subtype (88-12/88L, 88/DRB1, 12/88L-DRB1) of the three locus-haplotype 88-12/88L-DRB1.
Homozygous haplotypes	Two identical haplotypes in diploid cells.
Heterozygous haplotypes	Two different haplotypes in diploid cells.
Mongrels	Mixtures of different pure dog breeds or interbreeds.
