# Supplementary material for: Large-Scale Polymorphism Analysis of Dog Leukocyte Antigen Class I and Class II Genes (DLA-88, DLA-12/88L and DLA-DRB1) and Comparison of the Haplotype Diversity between Breeds in Japan
Source: Cells. 2023 Mar 6;12(5):809. doi: 10.3390/cells12050809 (PMC10001263; doi:10.3390/cells12050809)
Supplement: Supplementary file 1 [file cells-12-00809-s001.zip › Supplementary table S1.pdf]

Supplementary table S1. Information on primers using this study

Locus or allele	Primer name	Primer sequence (5' to 3')	Primer location	Annealing temperature	Product size	Reference
**A. 1st PCR primers for genotyping of *DLA-88, DLA-88L* and *DLA-12* genes**						
DLA-88	88-seg-F	AGGGGACAATGGGACAGGAACTTGGA	5' side of exon 1	63˚C	4.0 kb	(1)
	88-seg-R2	AGGACTCAGGGAGACAGTGCGACAAGA	3' side of exon 8			This study
DLA-88L and *DLA-12*	88L/12-seg-F	CATTTCTGTTGGGATATGTGGTAA	5' side of exon 1	58˚C	5.6 kb	(2)
	88L/12-seg-R	CATCAAGGGATAAGGTGAAAGA	3' side of exon 8			
**B. 2nd PCR primers for genotyping of *DLA-88L* and *DLA-12* genes**						
DLA-88L	88/88L-F	CGGAGATGGAGGTGGTGA	5' UTR and exon 1	63˚C	1.6 kb	(3)
	88/12/88L-R	GGTGGCGGGTCACACG	exon 4			
DLA-12	12-F	CGACCCTAAAGGTCTGGGCTA	5' UTR and exon 1	63˚C	1.6 kb	(2)
	88/88L/12-R	GGTGGCGGGTCACACG	exon 4			(3)
**C. RT-PCR and sequencing primers for genotyping of *DLA-DRB1* gene for mRNA**						
DLA-DRB1	DRB1-F	GCACCCTGTCCTTTCTG	exon 1	60˚C	797 bp	(4)
	DRB1-R	TCACCATCTCCACTTCAG	exon 8			
**D. primers for genotyping of *DLA-DRB1* gene for genomic DNA**						
DLA-DRB1	DRB1-g-F	CCGTCCCCACAGCACATTTC	intron 1 and exon 2	60˚C	322 bp	(5)
	DRB1-g-R	TGTGTCACACACCTCAGCACC	intron 2			
**E. Sequencing primers for genotyping of *DLA-88, DLA-88L* and *DLA-12 genes***						
DLA-88, DLA-88L and *DLA-12*	i1F-T	AGGGGGTCGGGCGGGGT	intron 1	50˚C		This study
DLA-88, DLA-88L and *DLA-12*	i3R-T	GAGTCCATATTCCCTTCCTGG	intron 3	50˚C		This study
DLA-88, DLA-88L and DLA-12	i2F2	GGTTTTACTTTCTCTTTGGACTG	intron 2	50˚C		This study

(1) Miyamae et al, Immunogenetics, 2021. (2) Miyamae *et al*, Immunogenetics, 2018. (3) Ross *et al*, Tissue Antigens, 2012. (4) Miyamae *et al*, Immunogenetics, 2019. (5) Wagner *et al*, 1996.
