# Supplementary material for: Large-Scale Polymorphism Analysis of Dog Leukocyte Antigen Class I and Class II Genes (DLA-88, DLA-12/88L and DLA-DRB1) and Comparison of the Haplotype Diversity between Breeds in Japan
Source: Cells. 2023 Mar 6;12(5):809. doi: 10.3390/cells12050809 (PMC10001263; doi:10.3390/cells12050809)
Supplement: Supplementary file 1 [file cells-12-00809-s001.zip › Supplementary table S2.pdf]

Supplementary table S2. Detailed information on each allele detected in this study.

Allele name	Number of alleles	Allele frequency (%)	Number of dogs	Novelly detected in this study	Local allele name	IPD-MHC Accession Number	NCBI database			
							Accession number	Sequence length (bp)	Determined region	Note
**DLA-88**										
DLA-88*001:03	41	2.47	31			DLA08261	LC462762	4,512	Full length of genomic region	
DLA-88*002:01	45	2.71	42			DLA08080	LC462763	4,494	Full length of genomic region	
DLA-88*003:02	142	8.56	118			DLA08269	LC462764	4,542	Full length of genomic region	Accession num. of full lengths of mRNA: LC462829
DLA-88*003:03	12	0.72	11		DLA-88*nov1	DLA08299	LC462778	4,543	Full length of genomic region	
DLA-88*004:02	128	7.72	104			DLA08113	LC462765	4,533	Full length of genomic region	
DLA-88*004:03	2	0.12	2		DLA88-402*	-	KX686902	746	exon 2 - intron 2 - exon 3	
DLA-88*005:01	56	3.38	51			DLA07975	LC462766	4,508	Full length of genomic region	
DLA-88*006:01	148	8.93	127			DLA07988	LC462767	4,529	Full length of genomic region	
DLA-88*006:02	11	0.66	11		DLA-88*nov3	DLA08300	LC462808	765	exon 2 - intron 2 - exon 3	
DLA-88*007:02	2	0.12	2		DLA-88*nov39	DLA08312	LC171433	546	exon 2 - exon 3 (CDS)	
DLA-88*008:02	2	0.12	2		DLA-88*nov71	-	LC592281	746	exon 2 - intron 2 - exon 3	exon 2 - exon 3 sequece is identical with DLA-88*00801m (MK617587)
DLA-88*010:01	10	0.60	10			DLA08063	LC592280	746	exon 2 - intron 2 - exon 3	
DLA-88*011:01	2	0.12	1			DLA08238	LC462768	4,508	Full length of genomic region	
DLA-88*012:01	74	4.46	67			DLA08165	LC462769	4,525	Full length of genomic region	
DLA-88*012:02	20	1.21	18			DLA08177	KR154222	765	exon 2 - intron 2 - exon 3	
DLA-88*013:02	49	2.96	35		DLA-88*nov7	DLA08305	LC462780	4,516	Full length of genomic region	
DLA-88*014:01:02	25	1.51	19		DLA-88*nov8	DLA08288	LC462781	4,541	Full length of genomic region	
DLA-88*019:02	16	0.97	13		DLA-88*nov10	DLA08297	LC462782	4,544	Full length of genomic region	
DLA-88*021:01	1	0.06	1			DLA08278	-	-	-	
DLA-88*021:02	8	0.48	8		DLA-88*nov11	DLA08317	LC130521	546	exon 2 - exon 3 (CDS)	
DLA-88*022:01	1	0.06	1			DLA08086	-	-	-	
DLA-88*025:01	15	0.90	13			DLA08089	LC462803	746	exon 2 - intron 2 - exon 3	
DLA-88*028:01	71	4.28	58			DLA08093	LC462770	4,543	Full length of genomic region	
DLA-88*028:03	13	0.78	10			DLA08205	LC271133	4,519	Full length of genomic region	
DLA-88*028:05	22	1.33	22		DLA-88*nov15	DLA08294	LC462813	746	exon 2 - intron 2 - exon 3	
DLA-88*028:06	1	0.06	1		DLA-88*nov32	DLA08308	LC171424	546	exon 2 - exon 3 (CDS)	
DLA-88*028:07	4	0.24	4		DLA-88*nov42	DLA08313	LC462821	746	exon 2 - intron 2 - exon 3	
DLA-88*032:01	11	0.66	11			DLA08194	LC462804	746	exon 2 - intron 2 - exon 3	1 nucleotide variation at intron 2 from KF911091 in NCBI database
DLA-88*032:02	1	0.06	1			DLA08204	KF911096	746	exon 2 - intron 2 - exon 3	
DLA-88*034:01	31	1.87	25			DLA08097	LC462772	4,544	Full length of genomic region	
DLA-88*035:01	21	1.27	16			DLA08098	LC462805	746	exon 2 - intron 2 - exon 3	
DLA-88*036:01	3	0.18	3			DLA08198	KF911094	746	exon 2 - intron 2 - exon 3	
DLA-88*036:02	15	0.90	13		DLA-88*nov18	DLA08291	LC462784	4,544	Full length of genomic region	
DLA-88*038:01	9	0.54	8			DLA08100	LC462806	746	exon 2 - intron 2 - exon 3	
DLA-88*039:01	6	0.36	6			DLA08101	-	-	-	
DLA-88*040:01	5	0.30	5			DLA08135	-	-	-	
DLA-88*041:01	13	0.78	12			DLA08103	LC462773	4,544	Full length of genomic region	
DLA-88*043:02	2	0.12	2		DLA-88*nov22	DLA08284	LC130524	546	exon 2 - exon 3 (CDS)	
DLA-88*043:04	13	0.78	13		DLA-88*nov21	DLA08292	LC462785	4,518	Full length of genomic region	
DLA-88*045:01	19	1.15	19			DLA08237	-	-	-	
DLA-88*046:02	1	0.06	1	○	DLA-88*nov47	-	LC458800	746	exon 2 - intron 2 - exon 3	
DLA-88*049:01	3	0.18	3			DLA08168	-	-	-	
DLA-88*049:02	20	1.21	20		DLA-88*nov24	DLA08301	LC462815	746	exon 2 - intron 2 - exon 3	
DLA-88*050:02	3	0.18	3	○	DLA-88*nov56	-	LC458809	746	exon 2 - intron 2 - exon 3	
DLA-88*051:01	53	3.20	45			DLA08206	KT350388	746	exon 2 - intron 2 - exon 3	
DLA-88*052:01	11	0.66	10		DLA-88*nov4	DLA08287	LC462779	4,512	Full length of genomic region	
DLA-88*052:02	10	0.60	10		DLA-88*nov31	DLA08307	LC462819	746	exon 2 - intron 2 - exon 3	
DLA-88*053:01	8	0.48	8		DLA-88*nov14	DLA08289	LC130506	546	exon 2 - exon 3 (CDS)	
DLA-88*054:01	31	1.87	28		DLA-88*nov17	DLA08290	LC462783	4,544	Full length of genomic region	
DLA-88*055:01	1	0.06	1		DLA-88*nov29	DLA08293	LC171421	546	exon 2 - exon 3 (CDS)	
DLA-88*056:01	9	0.54	9		DLA-88*nov9	DLA08295	LC462811	746	exon 2 - intron 2 - exon 3	
DLA-88*057:01	10	0.60	10		DLA-88*nov2	DLA08296	LC462807	746	exon 2 - intron 2 - exon 3	
DLA-88*058:01	16	0.97	16		DLA-88*nov23	DLA08298	LC462786	4,529	Full length of genomic region	
DLA-88*060:01	3	0.18	3		DLA-88*nov5	DLA08303	LC462809	746	exon 2 - intron 2 - exon 3	
DLA-88*060:02	11	0.66	7		DLA-88*nov6	DLA08304	LC462810	746	exon 2 - intron 2 - exon 3	
DLA-88*060:03	3	0.18	3	○	DLA-88*nov57	-	LC458810	546	exon 2 - exon 3 (CDS)	
DLA-88*061:01	5	0.30	5		DLA-88*nov28	DLA08306	LC462817	746	exon 2 - intron 2 - exon 3	
DLA-88*062:01	6	0.36	6		DLA-88*nov33	DLA08309	LC462820	765	exon 2 - intron 2 - exon 3	
DLA-88*063:01	1	0.06	1		DLA-88*nov34	DLA08310	LC171426	546	exon 2 - exon 3 (CDS)	
DLA-88*064:01	1	0.06	1		DLA-88*nov45	DLA08311	LC171429	546	exon 2 - exon 3 (CDS)	
DLA-88*065:01	3	0.18	3		DLA-88*nov46	DLA08314	LC462823	746	exon 2 - intron 2 - exon 3	
DLA-88*073:01	2	0.12	2	○	DLA-88*nov52	-	LC458805	746	exon 2 - intron 2 - exon 3	
DLA-88*078:01	1	0.06	1	○	DLA-88*nov48	-	LC458801	746	exon 2 - intron 2 - exon 3	
DLA-88*501:01	139	8.38	122			DLA08108	LC462775	4,514	Full length of genomic region	Accession num. of full lengths of mRNA: LC462828
DLA-88*501:02	1	0.06	1		DLA-88*nov41	DLA08354	LC171435	549	exon 2 - exon 3 (CDS)	
DLA-88*502:01	46	2.77	36			DLA08109	LC462776	4,518	Full length of genomic region	
DLA-88*508:01	111	6.69	101			DLA08116	LC462777	4,517	Full length of genomic region	
DLA-88*508:02	1	0.06	1			DLA08244	KF911092	749	exon 2 - intron 2 - exon 3	
DLA-88*508:03	7	0.42	7		DLA-88*nov37	DLA08357	LC462788	4,517	Full length of genomic region	
DLA-88*510:01	3	0.18	3		DLA-88*nov26	DLA08355	LC171419	549	exon 2 - exon 3 (CDS)	
DLA-88*511:01	38	2.29	38		DLA-88*nov25	DLA08356	LC462787	746	Full length of genomic region	
DLA-88*nov20	1	0.06	1			-	LC130520	546	exon 2 - exon 3 (CDS)	
DLA-88*nov27	5	0.30	5			-	LC462816	749	exon 2 - intron 2 - exon 3	
DLA-88*nov35	1	0.06	1			-	LC171427	546	exon 2 - exon 3 (CDS)	
DLA-88*nov38	1	0.06	1			-	LC171432	546	exon 2 - exon 3 (CDS)	
DLA-88*nov40	1	0.06	1			-	LC171434	546	exon 2 - exon 3 (CDS)	
DLA-88*nov43	1	0.06	1			-	LC171437	546	exon 2 - exon 3 (CDS)	
DLA-88*nov49	1	0.06	1	○		-	LC458802	749	exon 2 - intron 2 - exon 3	
DLA-88*nov50	1	0.06	1	○		-	LC458803	745	exon 2 - intron 2 - exon 3	
DLA-88*nov51	1	0.06	1	○		-	LC458804	746	exon 2 - intron 2 - exon 3	
DLA-88*nov53	1	0.06	1	○		-	LC458806	746	exon 2 - intron 2 - exon 3	
DLA-88*nov54	2	0.12	2	○		-	LC458807	749	exon 2 - intron 2 - exon 3	
DLA-88*nov55	1	0.06	1	○		-	LC458808	765	exon 2 - intron 2 - exon 3	
DLA-88*nov58	1	0.06	1	○		-	LC458811	746	exon 2 - intron 2 - exon 3	
DLA-88*nov59	3	0.18	3	○		-	LC458812	746	exon 2 - intron 2 - exon 3	
DLA-88*nov60	3	0.18	3	○		-	LC458813	746	exon 2 - intron 2 - exon 3	
DLA-88*nov61	3	0.18	3	○		-	LC458814	746	exon 2 - intron 2 - exon 3	
DLA-88*nov68	2	0.12	2	○		-	LC581758	745	exon 2 - intron 2 - exon 3	
DLA-88*nov70	1	0.06	1	○		-	LC581760	746	exon 2 - intron 2 - exon 3	
**DLA-88L**										
DLA-88*016:03	22	1.33	19			DLA08253	KF939645	746	exon 2 - intron 2 - exon 3	
DLA-88*016:04	18	1.09	15			DLA08224	LC462791	5,541	Full length of genomic region	
DLA-88*016:05	11	0.66	11		DLA-88*nov19	DLA08319	LC462814	746	exon 2 - intron 2 - exon 3	
DLA-88*017:01	142	8.56	117			DLA08082	LC189199	5,538	Full length of genomic region	Accession num. of full lengths of mRNA: LC462830
DLA-88*024:02	12	0.72	12		DLA-88*nov13	-	LC462812	746	exon 2 - intron 2 - exon 3	
DLA-88*024:03	16	0.97	12		DLA-88*nov12	-	LC462792	5,555	Full length of genomic region	
DLA-88*026:02	2	0.12	2		DLA-88*nov44	-	LC462822	745	exon 2 - intron 2 - exon 3	
DLA-88*029:01	111	6.69	95			DLA08156	LC271133	5,532	Full length of genomic region	
DLA-88*029:02	1	0.06	1		DLA-88*nov36	-	LC171430	546	exon 2 - exon 3 (CDS)	
DLA-88*042:02	2	0.12	2			DLA08286	KT337312	746	exon 2 - intron 2 - exon 3	
DLA-88*067:01	3	0.18	2	○	DLA-88*nov63	-	LC458816	746	exon 2 - intron 2 - exon 3	
DLA-88*067:02	1	0.06	1	○	DLA-88*nov62	-	LC458815	746	exon 2 - intron 2 - exon 3	
DLA-88*nov30	1	0.06	1			-	LC462818	746	exon 2 - intron 2 - exon 3	
DLA-88*nov64	1	0.06	1	○		-	LC458817	741	exon 2 - intron 2 - exon 3	
DLA-88*nov65	1	0.06	1	○		-	LC458818	815	exon 2 - intron 2 - exon 3	
DLA-88*nov66	3	0.18	3	○		-	LC458819	746	exon 2 - intron 2 - exon 3	
DLA-88*nov67	1	0.06	1	○		-	LC458820	746	exon 2 - intron 2 - exon 3	
DLA-88*nov69	2	0.12	2	○		-	LC581759	744	exon 2 - intron 2 - exon 3	
**DLA-12**										
DLA-12*001:01:01	747	45.05	545		DLA-12*1	DLA08375	LC462793	5,608	Full length of genomic region	Accession num. of full lengths of mRNA: LC462831
DLA-12*001:01:02	5	0.30	5		DLA-12*nov1-2	DLA08376	LC462824	817	exon 2 - intron 2 - exon 3	
DLA-12*001:01:03	127	7.66	113		DLA-12*nov1-3	DLA08377	LC462794	5,822	Full length of genomic region	
DLA-12*001:01:04	41	2.47	32		DLA-12*nov1-4	DLA08378	LC462825	817	exon 2 - intron 2 - exon 3	
DLA-12*001:03	38	2.29	35		DLA-12*nov3	DLA08379	LC462796	5,608	Full length of genomic region	
DLA-12*001:04:01	42	2.53	38		DLA-12*nov4	DLA08381	LC462798	5,606	Full length of genomic region	
DLA-12*001:04:02	2	0.12	1		DLA-12*nov4-2	DLA08393	LC130535	611	exon 1 - exon 3 (CDS)	
DLA-12*001:05	25	1.51	19		DLA-12*nov5	DLA08382	LC462799	5,604	Full length of genomic region	
DLA-12*001:06	1	0.06	1		DLA-12*nov16	DLA08390	LC130547	611	exon 1 - exon 3 (CDS)	
DLA-12*002:01	6	0.36	6		DLA-12*nov7	DLA08383	LC130538	611	exon 1 - exon 3 (CDS)	
DLA-12*002:02	34	2.05	29		DLA-12*nov8	DLA08384	LC462801	5,603	Full length of genomic region	
DLA-12*002:03	67	4.04	58		DLA-12*nov6	DLA08388	LC462800	5,603	Full length of genomic region	
DLA-12*002:04	55	3.32	49		DLA-12*nov2	DLA08389	LC462795	5,609	Full length of genomic region	
DLA-12*003:01	48	2.90	34		DLA-12*nov10	DLA08386	LC462826	816	exon 2 - intron 2 - exon 3	
DLA-12*004:01	20	1.21	19		DLA-12*nov9	DLA08385	LC462802	5,612	Full length of genomic region	
DLA-12*005:01	12	0.72	12		DLA-12*nov11	DLA08387	LC130542	611	exon 1 - exon 3 (CDS)	
DLA-12*nov12	1	0.06	1			-	LC130543	611	exon 1 - exon 3 (CDS)	
DLA-12*nov13	6	0.36	5			-	LC462827	817	exon 2 - intron 2 - exon 3	
DLA-12*nov14	3	0.18	3			-	LC130545	611	exon 1 - exon 3 (CDS)	
DLA-12*nov15	1	0.06	1			-	LC130546	611	exon 1 - exon 3 (CDS)	
DLA-12*nov17	3	0.18	3	○		-	LC458821	816	exon 2 - intron 2 - exon 3	
DLA-12*nov18	21	1.27	16	○		-	LC458822	815	exon 2 - intron 2 - exon 3	
DLA-12*nov19	1	0.06	1	○		-	LC458823	818	exon 2 - intron 2 - exon 3	
DLA-12*nov20	1	0.06	1	○		-	LC581761	817	exon 2 - intron 2 - exon 3	
DLA-12*nov21	1	0.06	1	○		-	LC581762	816	exon 2 - intron 2 - exon 3	
**DLA-DRB1**										
DRB1*001:01	133	8.02	123			DLA08021	LC495499	797	exon 1 - exon 6 (CDS)	
DRB1*001:02	43	2.59	33			DLA08022	NM_001014768	1,165	Full length of mRNA	
DRB1*001:03	1	0.06	1			DLA04849	-	-	-	
DRB1*001:06	1	0.06	1			DLA08212	LC496089	797	exon 1 - exon 6 (CDS)	
DRB1*002:01	104	6.27	81			DLA08024	LC495500	797	exon 1 - exon 6 (CDS)	
DRB1*002:03	23	1.39	21			DLA04850	LC495501	797	exon 1 - exon 6 (CDS)	
DRB1*002:04	9	0.54	8		DRB1*095:01	DLA04908	LC495542	797	exon 1 - exon 6 (CDS)	
DRB1*003:02	11	0.66	11			DLA04851	LC495502	797	exon 1 - exon 6 (CDS)	
DRB1*004:01	3	0.18	3			DLA08027	LC528234	797	exon 1 - exon 6 (CDS)	
DRB1*005:01	3	0.18	3			DLA08028	LC495503	797	exon 1 - exon 6 (CDS)	
DRB1*006:01	204	12.30	160			DLA08029	LC495504	797	exon 1 - exon 6 (CDS)	
DRB1*006:03	4	0.24	3			DLA08242	LC495505	797	exon 1 - exon 6 (CDS)	
DRB1*008:01	1	0.06	1			DLA08031	LC495506	797	exon 1 - exon 6 (CDS)	
DRB1*008:02	11	0.66	11			DLA08032	LC495507	797	exon 1 - exon 6 (CDS)	
DRB1*009:01	146	8.81	125			DLA08033	LC495508	797	exon 1 - exon 6 (CDS)	
DRB1*009:02	1	0.06	1			DLA08239	-	-	-	
DRB1*010:01:1	11	0.66	8			DLA08034	LC495509	797	exon 1 - exon 6 (CDS)	
DRB1*011:01	23	1.39	22			DLA08036	LC495510	797	exon 1 - exon 6 (CDS)	
DRB1*011:03	13	0.78	10			DLA04853	LC495511	797	exon 1 - exon 6 (CDS)	
DRB1*012:01	142	8.56	111			DLA08037	LC495512	797	exon 1 - exon 6 (CDS)	
DRB1*012:03	1	0.06	1		*novL	DLA08047	LC494674	797	exon 1 - exon 6 (CDS)	
DRB1*013:01	36	2.17	33			DLA08038	LC495513	797	exon 1 - exon 6 (CDS)	
DRB1*015:01	232	13.99	196			DLA08040	LC495514, LC495515, LC495516	797	exon 1 - exon 6 (CDS)	different 3 alleles with variation in exon 1
DRB1*015:02	145	8.75	126			DLA08041	LC495517	797	exon 1 - exon 6 (CDS)	
DRB1*015:03	9	0.54	9			DLA08043	LC495518	797	exon 1 - exon 6 (CDS)	
DRB1*015:04	2	0.12	2			DLA04855	LC495519	797	exon 1 - exon 6 (CDS)	
DRB1*015:05	4	0.24	4			DLA08133	LC495520	797	exon 1 - exon 6 (CDS)	
DRB1*016:01	5	0.30	5			DLA08044	LC495521	797	exon 1 - exon 6 (CDS)	
DRB1*017:01	3	0.18	3			DLA08045	LC495522	797	exon 1 - exon 6 (CDS)	
DRB1*017:02	4	0.24	4			DLA04856	LC495523	797	exon 1 - exon 6 (CDS)	
DRB1*018:01	25	1.51	23			DLA08046	LC495524	797	exon 1 - exon 6 (CDS)	
DRB1*020:01	47	2.83	42			DLA08048	LC495525	797	exon 1 - exon 6 (CDS)	
DRB1*023:01	30	1.81	24			DLA08051	LC495526	797	exon 1 - exon 6 (CDS)	
DRB1*024:01	3	0.18	3			DLA08052	-	-	-	
DRB1*025:01	25	1.51	19			DLA08053	LC495527	797	exon 1 - exon 6 (CDS)	
DRB1*028:01	2	0.12	2			DLA08056	LC495528	797	exon 1 - exon 6 (CDS)	
DRB1*029:01	4	0.24	3			DLA08057	LC495529	797	exon 1 - exon 6 (CDS)	
DRB1*032:01	3	0.18	3			DLA08060	-	-	-	
DRB1*033:01:1	11	0.66	9			DLA08061	LC495530	797	exon 1 - exon 6 (CDS)	
DRB1*032:01:2	1	0.06	1			DLA04860	-	-	-	
DRB1*036:02	5	0.30	5			DLA04861	LC130501	797	exon 1 - exon 6 (CDS)	
DRB1*040:01	13	0.78	9			DLA08068	LC495531	797	exon 1 - exon 6 (CDS)	
DRB1*046:01	26	1.57	18			DLA08074	LC495532	797	exon 1 - exon 6 (CDS)	
DRB1*048:01	17	1.03	15			DLA04863	LC495533	797	exon 1 - exon 6 (CDS)	
DRB1*048:04	2	0.12	1			DLA04911	LC495534	797	exon 1 - exon 6 (CDS)	
DRB1*052:01	12	0.72	11			DLA04869	LC495535	797	exon 1 - exon 6 (CDS)	
DRB1*056:01	45	2.71	33			DLA04873	LC495536	797	exon 1 - exon 6 (CDS)	
DRB1*058:01	3	0.18	3			DLA04875	LC495537	797	exon 1 - exon 6 (CDS)	
DRB1*073:01	11	0.66	11			DLA04885	LC495538	797	exon 1 - exon 6 (CDS)	
DRB1*075:01	2	0.12	2			DLA04887	LC495539	797	exon 1 - exon 6 (CDS)	
DRB1*077:01	1	0.06	1			DLA04889	-	-	-	
DRB1*092:01:1	19	1.15	17			DLA04905	LC495540	797	exon 1 - exon 6 (CDS)	
DRB1*094:01	5	0.30	5			DLA04907	LC495541	797	exon 1 - exon 6 (CDS)	
DRB1*DRB006	1	0.06	1			-	AB236368	270	exon 2	
*novA	1	0.06	1	○		-	LC130497	797	exon 1 - exon 6 (CDS)	
*novB	1	0.06	1			-	LC130499	797	exon 1 - exon 6 (CDS)	
*novC	3	0.18	3	○		-	LC130496	797	exon 1 - exon 6 (CDS)	
*novE	4	0.24	3	○		-	LC130498	797	exon 1 - exon 6 (CDS)	
*novF	2	0.12	2	○		-	LC130500	797	exon 1 - exon 6 (CDS)	
*novG	1	0.06	1	○		-	LC592278	270	exon 2	
*novK	5	0.30	5	○		-	LC592279	270	exon 2	
