# Supplementary material for: Large-Scale Polymorphism Analysis of Dog Leukocyte Antigen Class I and Class II Genes (DLA-88, DLA-12/88L and DLA-DRB1) and Comparison of the Haplotype Diversity between Breeds in Japan
Source: Cells. 2023 Mar 6;12(5):809. doi: 10.3390/cells12050809 (PMC10001263; doi:10.3390/cells12050809)
Supplement: Supplementary file 1 [file cells-12-00809-s001.zip › Supplementary table S3.pdf]

Supplementary table S3B. 59 Single 88-12/88L-DRB1haplotypes detected in this study

Single Hp ID.	Breed	*DLA-88**	*DLA-12/88L*		*DLA-DRB1**
			*DLA-12**	*DLA-88L (DLA-88*)*	
S1	American Cocker Spaniel	*028:01*	-	*029:01*	*006:01*
S2	Beagle	*508:02*	*001:01:03*	-	*002:01*
S3	Beagle	*061:01*	-	*024:02*	*001:01*
S4	Boston Terrier	*006:01*	*001:01:01*	-	*002:01*
S5	Boston Terrier	*034:01*	*002:03*	-	*004:01*
S6	Boston Terrier	*508:01*	*001:01:03*	-	*novA*
S7	Bulldog	*028:01*	-	*029:01*	*012:01*
S8	Bulldog	*028:01*	-	*029:01*	*novG*
S9	Chihuahua	*025:01*	-	*016:03*	*073:01*
S10	Collie	*052:02*	*001:01:04*	-	*002:01*
S11	Collie	*nov49*	-	*016:04*	*013:01*
S12	English Cocker Spaniel	*006:01*	*001:01:01*	-	*012:01*
S13	FrenchBulldog	*028:01*	-	*nov67*	*015:02*
S14	GermanShepherddog	*022:01*	*001:01:01*	-	*001:01*
S15	GoldenRetriever	*021:01*	-	*016:04*	*001:01*
S16	GoldenRetriever	*038:01*	*001:01:01*	-	*003:02*
S17	GoldenRetriever	*nov70*	-	*026:02*	*015:01*
S18	GreatPyrenees	*032:02*	*001:01:01*	-	*015:01*
S19	Husky	*508:01*	*001:01:03*	-	*040:01*
S20	Husky	*057:01*	-	*016:05*	*001:03*
S21	ItalianGreyHound	*501:01*	*001:01:01*	-	*006:03*
S22	ItalianGreyHound	*046:02*	*0001:04:01*	-	*029:01*
S23	Jack Russell Terrier	*028:03*	-	*029:01*	*015:05*
S24	Jack Russell Terrier	*032:01*	*001:01:01*	-	*009:01*
S25	Kaninchen Dachshund	*045:01*	*001:01:01*	-	*003:02*
S26	Kooikerhondje	*nov43*	*001:01:01*	-	*015:03*
S27	LabradorRetriever	*004:02*	*001:01:01*	-	*012:01*
S28	Maltese	*003:02*	-	*017:01*	*003:02*
S29	Maltese	*052:02*	*nov20*	-	*002:04*
S30	Miniature Dachshund	*nov35*	*001:01:01*	-	*novB*
S31	MiniaturePinscher	*501:01*	*nov19*	-	*009:02*
S32	MiniatureSchnauzer	*005:01*	*003:01*	-	*020:01*
S33	MiniatureSchnauzer	*nov38*	*002:03*	-	*023:01*
S34	mongrel	*004:02*	*001:01:01*	-	*009:01*
S35	mongrel	*034:01*	*002:03*	-	*020:01*
S36	Papillon	*508:01*	*001:01:01*	-	*002:01*
S37	Pomeranian	*003:02*	-	*nov64*	*001:01*
S38	Pomeranian	*012:01*	*001:01:01*	-	*015:02*
S39	Pomeranian	*064:01*	-	*026:02*	*009:01*
S40	Pomeranian	*nov51*	*001:01:01*	-	*015:01*
S41	Pug	*028:01*	-	*029:01*	*015:01*
S42	Pug	*nov55*	*001:01:01*	-	*015:01*
S43	Pug	*nov58*	-	*nov65*	*015:01*
S44	Shetland Sheepdog	*nov50*	-	*017:01*	*002:01*
S45	Shiba	*063:01*	-	*016:05*	*015:01*
S46	Shiba	*nov40*	*001:01:04*	-	*001:01*
S47	Shiba	*501:02*	*001:06*	-	*015:01*
S48	Shih Tzu	*019:02*	-	*016:04*	*033:01:2*
S49	Shih Tzu	*nov53*	*001:01:01*	-	*077:01*
S50	Standard Poodle	*002:01*	*001:01:01*	-	*015:01*
S51	Standard Poodle	*078:01*	-	*067:02*	*015:01*
S52	Toy Poodle	*051:01*	*002:04*	-	*017:02*
S53	Toy Poodle	*508:01*	*001:01:03*	-	*018:01*
S54	Toy Poodle	*028:05*	-	*029:01*	*001:06*
S55	Toy Poodle	*028:05*	-	*029:02*	*015:02*
S56	Toy Poodle	*036:02*	*002:03*	-	*015:02*
S57	Toy Poodle	*055:01*	-	*nov30*	*017:02*
S58	Yorkshire Terrier	*004:02*	*nov15*	-	*006:01*
S59	Yorkshire Terrier	*501:01*	*001:04:01*	-	*015:01*
